# hicream: A flexible framework to identify significantly different regions in Hi-C data

**DOI:** 10.1093/bioinformatics/btag631

**Published:** 2026-08-25

**Authors:** Elise Jorge, Toby Dylan Hocking, Pierre Neuvial, Nathalie Vialaneix, Sylvain Foissac

**Affiliations:** GenPhySE, Univ Toulouse, Toulouse INP, ENVT, INRAE, Castanet-Tolosan 31326, France; LASSO lab, Département d’informatique, Université de Sherbrooke, Sherbrooke QC J1K-2R1, Canada; Institut de Mathématiques de Toulouse, UMR 5219, Université de Toulouse, CNRS UPS, Toulouse 31062, France; Université Fédérale de Toulouse, INRAE, MIAT, Castanet-Tolosan 31326, France; GenPhySE, Univ Toulouse, Toulouse INP, ENVT, INRAE, Castanet-Tolosan 31326, France

## Abstract

**Motivation:**

The three-dimensional (3D) organization of the genome plays a central role in many biological processes, and its disruption can critically impair cellular function. Detecting such changes is therefore crucial and can be achieved through differential analysis of Hi-C data. By comparing Hi-C data across biological conditions, differential Hi-C analysis aims to identify statistically significant and biologically relevant changes in chromatin organization. However, most existing methods either target a single predefined type of structure (e.g. TADs, loops, or compartments), thereby limiting their ability to uncover novel or complex structural variations, or produce highly local and disconnected results that are difficult to interpret in terms of higher-order genome organization.

**Results:**

We introduce *hicream*, a novel framework for differential Hi-C analysis that identifies regions of arbitrary shape, allowing to uncover new differential structures. By combining pixel-level differential analysis and data-driven clustering to define candidate regions of the Hi-C interaction matrix, our approach provides an interpretable measure of the differential signal for each region through a post hoc inference strategy. The resulting differential regions can be explored using an interactive visualization interface.

**Availability and Implementation:**

*hicream* is available at https://cran.r-project.org/package=hicream

## 1 Introduction

In eukaryotic cells, chromosomes are compacted within the nucleus and organized into three-dimensional (3D) structures at different levels, including chromosomal territories, chromatin compartments, topologically associating domains (TADs), and chromatin loops. This three-dimensional (3D) organization regulates gene expression and plays a crucial role in various biological processes such as cell division and differentiation (Oudelaar *et al.* 2020). It has been shown to be involved in cancers and congenital anomalies (Lupiáñez *et al*. 2015, Spielmann *et al*. 2018).

The 3D genome architecture can be profiled using Hi-C (Lieberman-Aiden *et al*. 2009), which quantifies interaction frequencies between genomic intervals (“bins”) along the chromatin. Hi-C data are commonly represented as sparse, symmetric interaction matrices, where each entry records the number of detected interactions between bins *i* and *j*, where (i, j) is called a “pixel.” By comparing such matrices across biological conditions, differential Hi-C analysis aims at identifying statistically significant and biologically relevant changes in chromatin organization (Gjoni *et al*. 2025).

A first class of approaches to differential analysis of Hi-C data focuses on structures and aims to identify differential regions of the Hi-C matrix corresponding to an *a priori* defined type of structure, such as chromatin loops (Lareau and Aryee 2018), TADs (Mourad 2022, Hua *et al*. 2024) or A/B compartments (Chakraborty *et al*. 2022, Maigné *et al*. 2025). This strategy constrains the analysis to one type of structure at a time, requiring to use multiple tools to capture variations across different levels of chromatin organization. Moreover, it either relies on rigid, structure-specific shape assumptions for differential regions, or is limited to detecting differential boundary locations. This leaves aside the diversity of shapes that structural variations can produce: For instance, Cresswell and Dozmorov (2020) identify several types of TAD changes with distinct differential region shapes, such as a triangle for TAD strengthening and a stripe for a boundary shift. Directly identifying differential regions in a shape-agnostic manner is therefore more flexible and comprehensive. Last, analyzing each structural type independently does not naturally account for complex chromatin variations arising from the interplay of multiple structural elements across different scales of organization.

A second class of approaches, referred to as “pixel-based” differential analysis, focuses on identifying differential pixels individually (Jorge *et al*. 2025b). For each pixel (i,j), a test is performed and a *P*-value is computed. A global multiple testing correction using the Benjamini-Hochberg (BH) procedure (Benjamini and Hochberg 1995) is then applied across all pixels to control the False Discovery Rate (FDR), defined as the expected proportion of false positives among the rejected hypotheses. The main limitation of this approach lies in the disconnected nature of the results and its dependence on matrix resolution. Restricting the analysis to independent pixels is not well suited to detecting structural variations that extend beyond regions of an arbitrary pixel size. To address this limitation, the *diffHic* package (Lun and Smyth 2015) introduced the *diffHic clustering* method, which aims to form clusters of significant pixels based on adjusted *P*-values. However, as noted in the package documentation, this procedure does not provide formal statistical guarantees, as it relies on a potentially biased estimate of the cluster-level FDR.

To overcome the limitations of these approaches, we introduce *hicream*, a data-driven framework for detecting genomic structural variations by identifying differential regions of arbitrary size and shape between groups of Hi-C matrices. *hicream* builds upon a pixel-based differential analysis followed by a clustering step. It then combines the resulting clusters and associated *P*-values with a *post hoc* statistical inference framework (Goeman and Solari 2011) to estimate the proportion of differential pixels within each cluster and to select clusters with the highest estimated proportions. We evaluated *hicream*, compared it against the *diffHic clustering* approach, and applied it to two independent real datasets, identifying differential regions. Results show consistency with external data, including active CTCF binding sites from ChIP-seq experiments and enriched transcription factor binding sites (TFBS).

## 2 Materials and methods

### 2.1 Differential analysis of Hi-C data

In this article, we address the comparison between two groups of $r=r_{1}+r_{2}$ Hi-C matrices, which are (p×p)-symmetric matrices, with nonnegative integer entries for *p* bins. These matrices are denoted $M^{g,l}$, where g∈{1,2} stands for the group to which the matrix belongs (also called the “condition”) and $l\in \{1,\ldots ,r_{g}$} corresponds to the *l*-th replicate of condition *g*. Note that these replicates are not assumed to be paired between conditions. The matrix entries are denoted by $m_{ij}^{g,l}$. They represent the interaction frequency (in terms of number of reads) between bins *i* and *j* for the *l*-th matrix of condition *g*. Note that we only consider intra-chromosomal interactions, *i.e*, matrices corresponding to a single chromosome.

In this setting, the objective of Hi-C data differential analysis is to identify changes within the 3D conformation of the genome between the two conditions g=1 and g=2, i.e. differential clusters of pixels.

### 2.2 Proposed workflow: hicream

**Figure 1:**
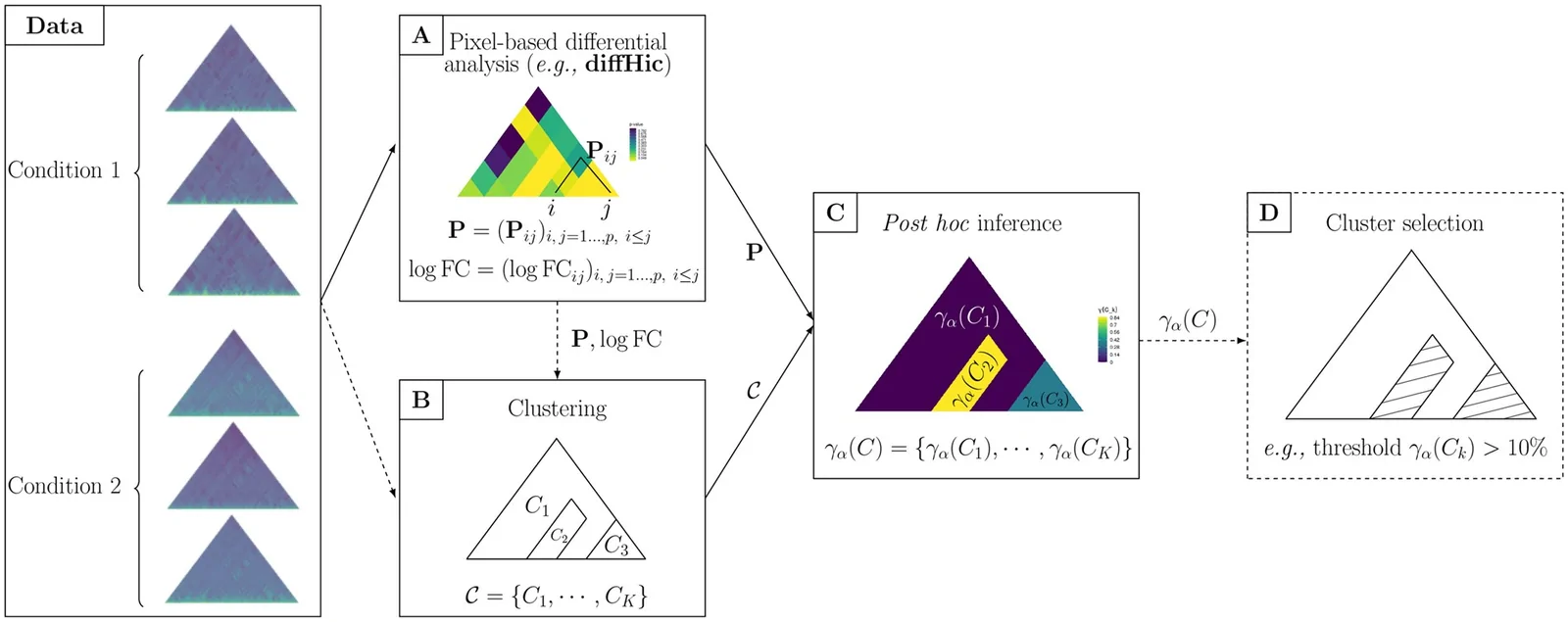
The hicream workflow. Input data correspond to Hi-C matrices from two different biological conditions, with several replicates in each. (A) A pixel-based differential analysis is performed (e.g. using *diffHic*), rendering a map of *P*-values where each *P*-value $P_{ij}$ corresponds to the test performed for pixel (i,j). (B) A clustering of the pixels is then obtained, providing *K* clusters of pixels $C=\{C_{1},\ldots ,C_{K}\}$. The clustering step can take as inputs either the Hi-C matrices—regardless of their condition—or the results of the pixel-level differential analysis. (C) The *post hoc* inference step takes as inputs the *P*-values computed at step (A) and the clusters of pixels defined at step (B) to compute $\gamma_{\alpha }(C_{k})$, the (minimal) TDP (True Discovery Proportion) for each cluster $C_{k}$. (D) An optional step of cluster selection can finally be performed by thresholding $\gamma_{\alpha }(C_{k})$.

Our approach is implemented in the R package *hicream*, following the workflow illustrated in Fig. 1.

- A statistical test is performed for each pixel to assess significant differences between conditions, resulting in a *p*-value map. We chose *diffHic* to perform this differential analysis, as explained in Section 2.2.1;
- A set of pixel clusters, $C=\{C_{1},C_{2},\ldots ,C_{K}\}$, is either obtained by an automated clustering method, or chosen by the user. Possible clustering choices are discussed in Section 2.2.2, including data-driven methods;
- The *p*-value map and the clusters are combined using a *post hoc* approach described in Section 2.2.3 to provide a lower bound on the true discovery proportion (TDP) in each cluster of C, ${\left(\gamma_{\alpha }(C_{k})\right)}_{k=1,\ldots ,K}$, with high probability 1−α;
- (optional) The user can define a TDP threshold to select some of these clusters (those with enough statistical evidence) for further interpretation (e.g. enrichment analyses).

These steps are described in the next sections and further information on the method is also provided in Supplementary Section S1.

#### 2.2.1 Pixel-based differential analysis

Our recent benchmark of pixel-based differential analysis methods (Jorge *et al*. 2025b) highlighted the wide diversity of available approaches and their heterogeneous statistical performance. Among them, the method implemented in the R/Bioconductor package *diffHic* (Lun and Smyth 2015) showed strong results and was therefore selected as the default option in *hicream*. For every pixel (i,j), it results in a *P*-value half-matrix, ${\left(P_{ij}\right)}_{i,\;j=1\ldots ,p,\;i\leq j}$, which contains the result of $m\leq \frac{p(p+1)}{2}$ tests performed at the pixel level, where *m* is the number of pixels for which at least one interaction has been observed in one of the *r* Hi-C matrices.

#### 2.2.2 Pixel clustering

The second step of the workflow consists of defining pixel clusters, $C=\{C_{1},C_{2},\ldots ,C_{K}\}$. Importantly, the *hicream* workflow can cope with any number of pixel clusters, including overlapping ones, without compromising its statistical guarantees. Users may therefore define regions based on prior knowledge or specific assumptions.

In the absence of such prior knowledge or expectations regarding regions of interest, *hicream* proposes an automatic, data-driven clustering method. This strategy identifies clusters of pixels that are similar either in terms of interaction counts (*hicream counts*) or with respect to the results of the differential analysis (*hicream diff*), while respecting the 2D organization of the Hi-C matrix. Both approaches rely on a constrained hierarchical clustering based on Ward’s linkage (Randriamihamison *et al*. 2021). This method takes as input a matrix X with *m* rows. It starts with an initial clustering where each pixel is a cluster and greedily agglomerates pairs of adjacent clusters that provide the smallest change in within-cluster inertia.

In *hicream counts*, for each of the *m* pixels q:=(i,j), X contains its (normalized by *diffHiC*) counts across replicates and conditions: $X_{q,.}=(m_{ij}^{1,1},\ldots ,m_{ij}^{1,r_{1}},m_{ij}^{2,1},\ldots ,m_{ij}^{2,r_{2}})$. In *hicream diff*, X uses the results of the differential analysis, aggregating the log-transformed adjusted *P*-values and the log-fold change values: $X_{q,.}=(\text{log}\;P_{ij}^{\text{adj}},{\text{logFC}}_{ij})$. The spatial dependence between adjacent pixels from the 2D Hi-C matrix is encoded in the connectivity graph G, where nodes *V* correspond to pixels and edges link adjacent pixels: Two pixels $\left(i_{1},j_{1}\right)$ and $\left(i_{2},j_{2}\right)$ are said to be adjacent if $\left(i_{2},j_{2})=(i_{1},j_{1}\pm 1\right)$ or $\left(i_{2},j_{2})=(i_{1}\pm 1,j_{1}\right)$. Further details on how missing data are handled in the connectivity graph are provided in Supplementary Section S1.

This approach gives a hierarchical clustering that can be represented as a dendrogram and is a collection of clusterings. The final clustering is selected by finding the elbow point of the Ward’s linkage.

In practice, we used the algorithm implemented in *scikit-learn* (Pedregosa *et al*. 2011) which is a greedy algorithm, iteratively merging two clusters while respecting the adjacency constraint. The final clustering was identified using the Python package *kneebow* that implements the elbow criterion. Other clustering strategies and model selection criteria could also be used within our framework.

#### 2.2.3 *Post hoc* inference

*Post hoc* inference methods (Goeman and Solari 2011) provide statistical guarantees for arbitrary sets of clusters. For a subset of pixels C⊂H:={1,…,m}, let TDP(C) be the proportion of true positives (or True Discovery Proportion) in *C*, that is, the (unknown) proportion of pixels (i,j) in *C* such that the null hypothesis H${\;}_{0,ij}$ is false. Given a target risk level α∈(0,1), *post hoc* inference builds, from the data, a function $\gamma_{\alpha }$ which assigns to each subset C⊆H a value $\gamma_{\alpha }(C)\in [0,1]$, such that


(1) $$P\left(\forall C\subset H,\;\text{TDP}(C)\geq \gamma_{\alpha }(C)\right)\geq 1-\alpha .$$

With probability at least 1−α, for any *C*, the proportion of true positives in *C* is at least $\gamma_{\alpha }(C)$. A function $\gamma_{\alpha }$ satisfying (1) is called a *post hoc bound* for the TDP.

While both FDR and *post hoc* inference provide statistical control on the proportion of errors (FDR=E(FDP) where FDP=1−TDP is the proportion of false discoveries), we emphasize that they provide fundamentally different statistical guarantees:

- First, while FDR statements are true on average (*in expectation*) over all possible experiments that could have been performed, *post hoc* inference statements are true *with probability* 1−α. For example, if for α=0.05 we find γ(C)=0.875 for a given cluster *C*, it means that we are 95% confident that more than 87.5% of the pixels in *C* are different between the two conditions.
- Second, standard FDR controlling procedures are not applicable to data-driven pixel clusters (Goeman and Solari 2011). In contrast, *post hoc* bounds provide statistical guarantees for any number of user-defined subset of hypotheses (here, pixel clusters).

Here, we use the Simes bound introduced by Goeman and Solari (2011) and defined as $\gamma_{\alpha }:C\;\mapsto \;1-V_{\alpha }(C)/|C|$, where


(2) $$V_{\alpha }(C)=\underset{1\leq n\leq |C|}{\text{min}}\left[\sum_{(i,j)\in C}\;1_{\left\{P_{ij}>\frac{\alpha n}{m}\right\}}+n-1\right].$$

The formulation of Equation (2) was obtained by Blanchard *et al*. (2020) and a linear time implementation is described in Enjalbert-Courrech and Neuvial (2022). It only requires the availability of a *P*-value $P_{ij}$ corresponding to positively dependent (PRDS) test statistics as defined in Benjamini and Yekutieli (2001). As such, it is valid under the same assumptions as the widely used BH procedure for FDR control.

### 2.3 Results evaluation

#### 2.3.1 Semi-synthetic data benchmark

We first evaluated *hicream* using semi-synthetic data with a controlled ground-truth signal, as done previously (Jorge *et al*. 2025b). Briefly, real Hi-C contact matrices from the same biological condition (for which no differences are expected) were used as a null hypothesis baseline. Artificial signals were then introduced into specific local regions of a subset of matrices to assess the capacity of the methods to detect them. More specifically, we used five technical replicates of a Hi-C library from a human colon sample (The ENCODE Project Consortium 2012). Four of the five technical replicates were used to form two artificial groups of duplicates, while the fifth replicate was used to add counts in predefined regions of the Hi-C matrices, creating known differences between the two groups. This process is illustrated in Fig. S2, available as supplementary data at *Bioinformatics* online.

We tested and compared the two versions of *hicream* and the approach proposed in *diffHic clustering*. Clusters of interest were then selected as:

- *hicream*: clusters for which the *post hoc* bound (for α=0.05) was larger than a given threshold (i.e. as in step (D) of Fig. 1);
- *diffHic clustering*: clusters for which the *cluster-level FDR* computed by the package was smaller than a given threshold.

These two threshold values are set independently of each other since the two criteria are not comparable.

For a given threshold and method, we obtained the precision as the proportion of pixels correctly declared differential among all pixels in selected clusters. The recall (power) was also computed similarly. Precision-Recall (PR) curves were obtained by varying the selection threshold from 5% to 100% for *diffHic clustering* and for each unique observed value of $\gamma_{\alpha }$ for *hicream*. A perfect classifier would yield a precision of 1 (no false positives) and a recall of 1 (no false negatives). Therefore, the “best” clustering for each method was obtained as the (Precision, Recall) point closest to (1,1) (which corresponds to two distinct thresholds for the two methods). The evaluation was carried out for three chromosomes (1, 7, and 21) at three resolutions (200 kb, 500 kb, and 1 Mb).

#### 2.3.2 Application to real use case datasets

The first dataset comes from a Hi-C study conducted on a murine cell line under two condition: either CTCF-depleted (CTCF- condition) or control (CTCF+ condition) (Zhang *et al*. 2021). Additional data from ChIP-seq experiments against CTCF on the same cell line were also used (Zhang *et al*. 2019). Hi-C matrices and ChIP-seq narrow peaks were downloaded from GEO (accessions GSE168251 and GSE129997).

The second real dataset comes from a comparison of mouse cell lines at different stages of neuronal differentiation (Bonev *et al*. 2017). Raw Hi-C data from stem cells (ES) and cortical neurons (CN) were downloaded from GEO (accession GSE96107) and processed with the *nfcore/hic* pipeline to obtain four matrices (replicates) of each condition at 50 kb resolution. Corresponding ChIP-seq data were obtained from the UCSC Table Browser.

Each dataset was processed with *hicream diff*. To better characterize the shape of obtained clusters and profile their geometrical diversity with respect to their TDP, we designed three shape-related metrics and assigned them to each cluster: a triangle-, a stripe-, and a square-shape score. Relationships between shape metrics, TDP, and absolute log-fold change (logFC) were characterized using Principal Component Analysis (PCA).

Then, clusters with a minimal TDP greater than 10% across all chromosomes were selected for further analysis.

Clusters selected in the neuronal differentiation study were compared with the results obtained from two other tools designed to identify significant differences in TADs (*TADCompare*, Cresswell and Dozmorov [2020]) or compartments (*dcHiC*, Chakraborty *et al*. [2022]). Unlike *hicream*, both tools identify 1D location on the genome corresponding to changes. Hence, selected *hicream* clusters were projected onto the genome to identify the corresponding genomic boundaries and their flanking bins. Then, to assess the consistency between *hicream* and *TADCompare*, we tested for the enrichment of these cluster boundaries in differential shifted regions identified by *TADCompare*, compared to flanking bins. As for *dcHiC*, we explored the relationship, at the pixel level, between TDP (related to the cluster in which the pixel is included) and major changes in compartment conformation (when a pixel is between two “A” regions in condition 1 and between two “B” regions in condition 2, or the opposite).

*hicream* clusters of both studies were also compared with known biological signal related to genomic organization: We tested for CTCF enrichment at cluster boundaries compared with the flanking bins, by using data on active CTCF binding sites from ChIP-seq experiments. In addition, the distribution of active CTCF binding sites was further characterized by profiling their relative positions with respect to the cluster boundaries.

Finally, cluster boundaries were also tested for enrichment of all known TFBSs from the JASPAR database (CORE Vertebrates 2024) using the AME tool from the MEME Suite (v5.5.4). Last, genes with enriched binding motifs from the TFBS analysis were also subjected to a Gene Ontology functional analysis using the Panther Classification System tool from https://geneontology.org (PANTHER GO-Slim Biological Process, Fisher exact test and Bonferroni multiple testing correction).

Further information on data processing and cluster validation is provided in Supplementary Section S2, available as supplementary data at *Bioinformatics* online.

### 2.4 Package implementation and visualization

*hicream* is implemented as an R package available on CRAN (Jorge *et al*. 2025a). Since the default plotting functions provide only static visualizations, we additionally developed an interactive interface based on the R package *animint2* (Sievert *et al*. 2019). All results from this study can be explored online at https://eoojj.github.io/hicream-article-viz/. A detailed illustration of this interactive visualization platform is provided in Fig. S3, available as supplementary data at *Bioinformatics* online.

## 3 Results

### 3.1 On semi-synthetic data, hicream identifies relevant clusters of differential pixels

**Figure 2:**
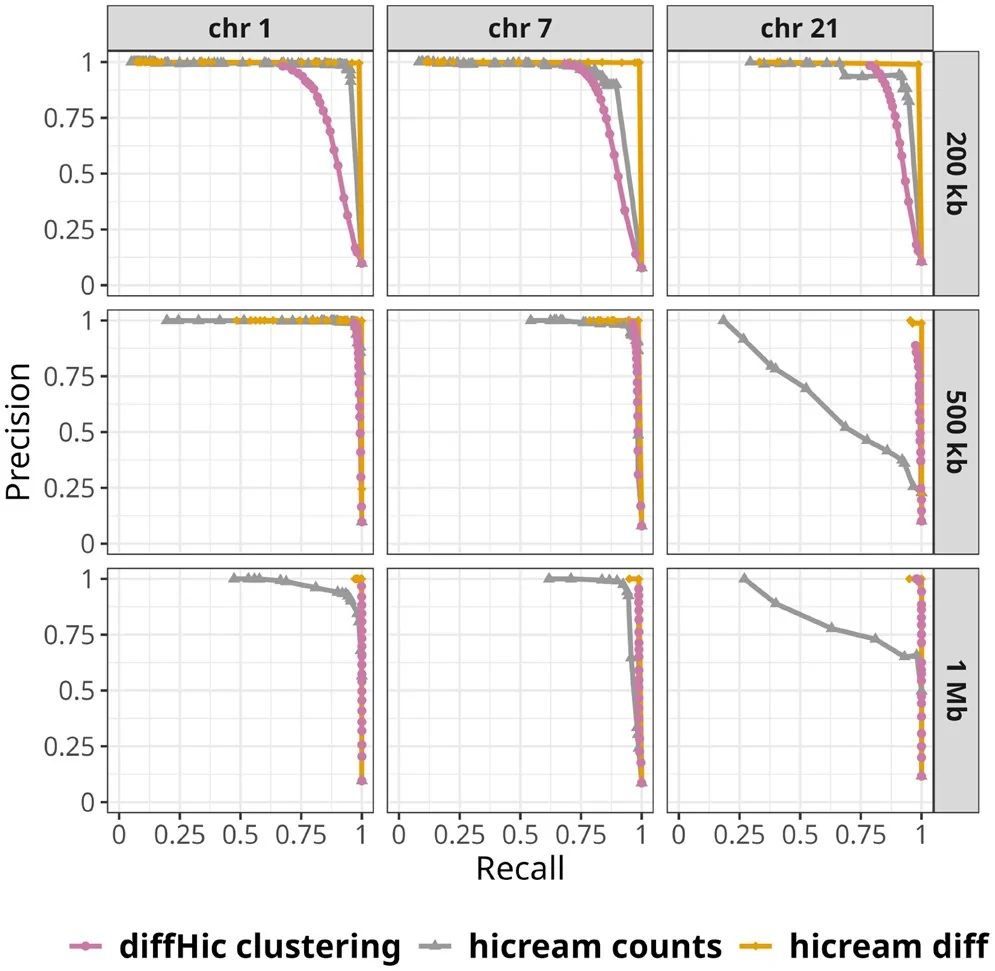
PR curves (semi-synthetic data) computed for three chromosomes of contrasted lengths (chr 1, 7, and 21), at three different bin resolutions (200 kb, 500 kb, and 1 Mb), and for the three methods (both *hicream* versions and *diffHic clustering*). Every point corresponds to a different given threshold value (see Section 2.3.1).

Figure 2 displays Precision-Recall curves for the three compared approaches (both *hicream* versions and *diffHic clustering*) on the semi-synthetic dataset. *hicream diff* systematically outperforms *diffHiC clustering* and *hicream counts*. *hicream counts* outperforms *diffHic clustering* at 200 kb resolution but does not show better performance at 500 kb or 1 Mb resolution. As the *hicream counts* variant does not outperform the default *hicream diff* method, we chose to focus on *hicream diff* only in the sequel.

Table 1 gives some characteristics of the “best” selection for each chromosome and resolution for *hicream diff* and *diffHic clustering*. Across all resolutions and chromosomes, *hicream diff* can reach high performance (in terms of precision and recall) with a small number of clusters compared to *diffHic clustering*. Indeed, as can be observed for chromosome 1 at 200 kb, *hicream diff* extracts only 120 clusters—118 times less than *diffHic clustering—*with a median size of 36 pixels, which is 12 times larger than *diffHic clustering*. Moreover, for chromosome 21 at 1 Mb, despite showing similar performance, both methods largely differ in their output clustering as *hicream diff* summarizes the results in only 7 clusters, while *diffHic clustering* yields 100 clusters, most of them being composed of only one single pixel. Altogether, these results highlight the agreggating and smoothing properties of *hicream*, which allows to properly cluster pixels, facilitating result interpretation.

**Table 1:** Characteristics of best performing clusterings (semi-synthetic data).

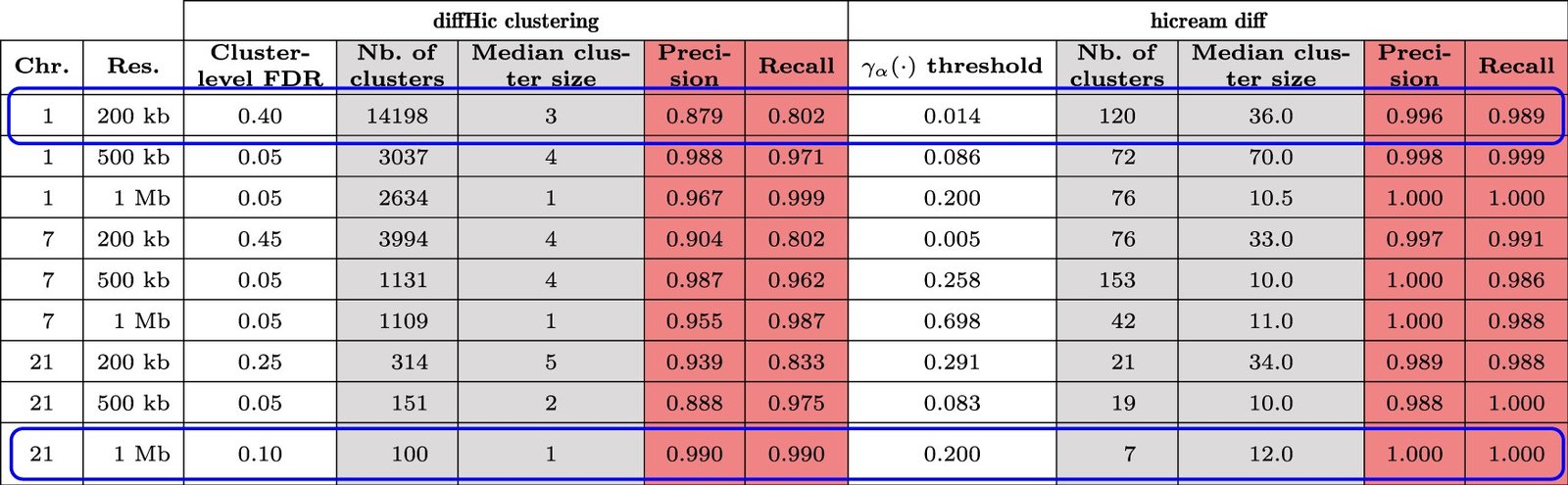

The best performance corresponds to the (precision, recall) point closest to (1,1). For all chromosomes and resolutions and for both *diffHic clustering* and *hicream diff*, information about clustering statistics (number of clusters and their median size in number of pixels) as well as corresponding precision and recall are provided. For *hicream diff*, *post hoc* inference was applied with α=0.05 and the threshold used for cluster selection is given in the column “$\gamma_{\alpha }(\cdot )$ threshold.” Two particular cases are circled in blue and commented in the text.

### 3.2 On real data, hicream identifies clear and contrasted clusters of various shapes

**Figure 3:**
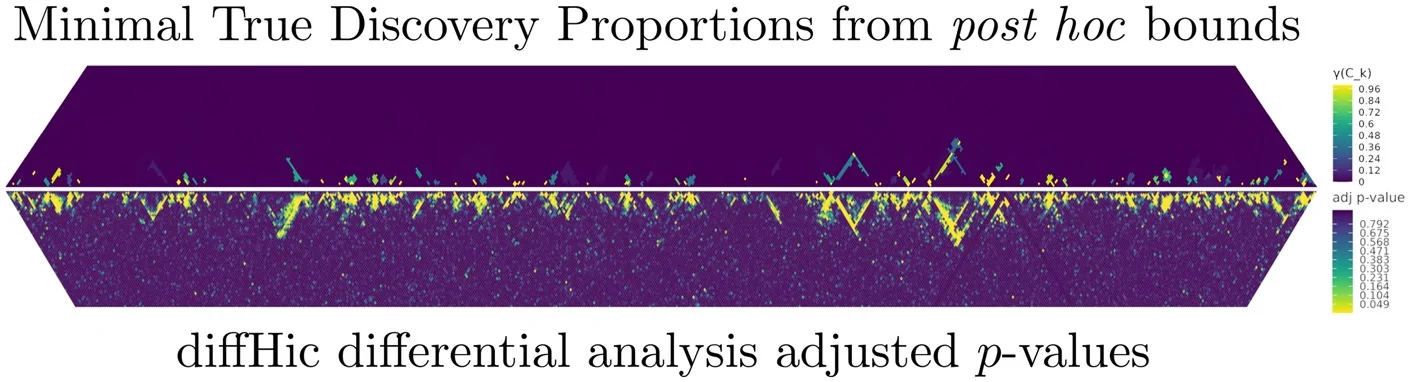
Illustration of hicream smoothing and aggregating properties (CTCF depletion study). *hicream diff* was applied to chromosome 13 of the CTCF depletion dataset at 200 kb resolution. The *kneebow* rule selected 1261 clusters. Upper half: *hicream diff* results. Each cluster of pixels $C_{k}$ is displayed with a color corresponding to its minimal proportion of true positives $\gamma (C_{k})$. Lower half: adjusted *P*-values obtained from *diffHic* differential analysis. The Hi-C matrix representation has been truncated to focus on the diagonal.

By applying *hicream* to real Hi-C datasets, we found that our method reveals sharp clusters from pixel-based results of differential analysis. Figure 3 illustrates these observations in the CTCF depletion data: the upper half displays the cluster-based minimal TDP obtained by *hicream diff*, while the lower half shows the original pixel-based adjusted *P*-values derived from *diffHic* (chromosome 13, 200 kb). High-TDP clusters in the upper half typically align with groups of pixels exhibiting significant adjusted *P*-values in the lower half, with sharper and more distinct boundaries. This contrast is improved by the fact that significant yet isolated pixels in the lower half usually get assigned to large clusters with low TDP bounds in the upper half. This illustrates how *hicream* can extend the results of *diffHic*, by removing the background noise, highlighting the most differential regions and providing statistical guarantees.

**Figure 4:**
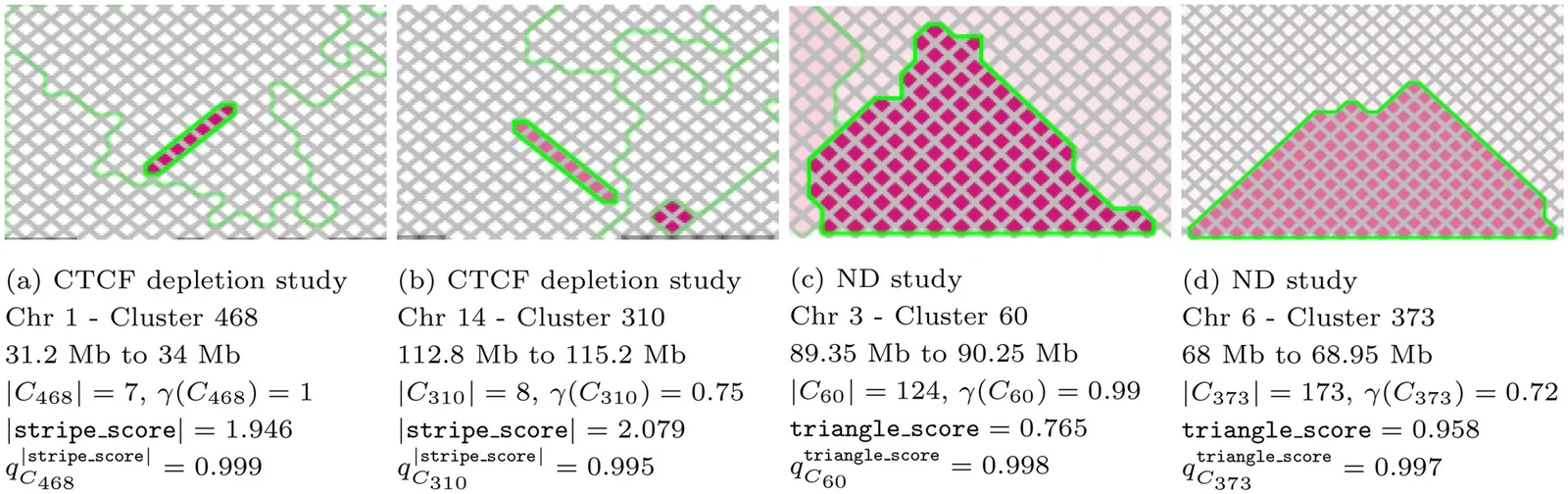
Stripe and triangle -shaped differential clusters identified by hicream (CTCF depletion and Neuronal Differentiation studies). The green lines are cluster boundaries and each cluster $C_{k}$ is colored according to $\gamma_{\alpha }(C_{k})$. Four selected clusters, highlighted with a slightly more vivid boundary color, are examples of stripe-shaped differential clusters identified with *hicream diff* in the CTCF depletion study at 200 kb resolution, and triangle-shaped differential clusters identified in the neuronal differentiation dataset at 50 kb resolution. For each cluster, information about chromosome, cluster number, cluster position on the chromosome (the left and right positions of the first and last chromosome bins, respectively, among the pixels belonging to this cluster), size (number of pixels), minimal True Discovery Proportion $\gamma_{\alpha }(C_{k})$, shape score (either stripe_score or triangle_score), and quantile of the shape score for the corresponding cluster is given below the plot.

Importantly, the resulting differential clusters exhibit a wide range of shapes and sizes, showing greater diversity than is typically observed in Hi-C interaction matrices (Figs 3 and 4). Whereas Hi-C data are generally characterized by a hierarchical TAD-like organization, the focused and precise delineation of groups of differentially proximal interactions makes it possible to highlight the variable components of these canonical structures. In this context, the ability of *hicream* to detect statistically significant clusters of arbitrary shape without imposing *a priori* constraints constitutes a valuable feature.

Figure S4, available as supplementary data at *Bioinformatics* online further illustrates the diversity of shapes identified by our method with a PCA of shape metrics, TDP and logFC. It exhibits substantially different patterns across biological datasets. Interestingly, on the CTCF depletion study, high TDPs are associated with large stripe-scores, consistent with the chromatin extrusion process underlying TAD boundary and chromatin loop formation. On the contrary, in the neuronal differentiation study, high TDPs are associated with large triangle-scores, showing that a different biological process (TAD compaction or de-compaction) is at hand in this dataset. Overall, this supports the notion that various patterns of 3D variation drive distinct functional effects, and suggests that *hicream* can capture a broad range of dynamic conformations that are not limited to a specific structure.

To confirm this generality of application, we compared *hicream* clusters with predictions from two structure-level differential methods, both run on the neuronal differentiation data: *TADCompare* to detect variable TAD boundaries, and *dcHiC* to detect differences in chromatin compartmentalization.

Figure S5, available as supplementary data at *Bioinformatics* online shows the enrichment of bins identified as differentially shifted by *TADCompare* and enriched in CN (resp. ES) condition at positive (resp. negative) log-fold change cluster boundaries. As a negative control, the same figure shows a depletion of differentially shifted bins enriched in CN (resp. ES) at negative (resp. positive) log-fold change cluster boundaries.

To compare with compartment differences, we extracted pixels found significantly involved in a major compartment change by *dcHiC*: These were pixels “AA2BB” (between two bins in “A” compartment for the ES condition and in “B” compartment for the CN condition) and “BB2AA” (that correspond to the opposite situation). Figure S6, available as supplementary data at *Bioinformatics* online shows the distribution of cluster TDP for these specific pixels compared to all other pixels and exhibits a significantly increased TDP for pixels corresponding to compartment changes (Wilcoxon test, *P*-values <2.22e−16).

The link between compartment differences and differential clusters identified by *hicream* is not limited to “AA2BB” changes only. Indeed, Fig. S7, available as supplementary data at *Bioinformatics* online displays a group of 7 differential clusters (TDP>10%) identified by *hicream* on chromosome 19 of the neuronal differentiation study that represent an “AA2AB” switch. The left projections of the 7 clusters are all located in a region of non-differential bins corresponding to the A compartment in both ES and CN conditions. On the other hand, the right projections of the clusters contain 3 differential bins that switch from A compartment in ES condition to B compartment in CN condition. Therefore, those clusters correspond to pixels representing AA interactions in ES condition and AB interactions in CN condition. This is consistent with the fact that the 7 clusters have a negative log-fold change.

These results confirm that although *hicream* is neither designed nor limited to find differences in TADs or compartments specifically, it is able to identify signal related to multiple levels of chromatin organization at once, showing consistency with structure-level prediction tools.

### 3.3 Differential clusters identified by hicream show relevant TFBS enrichment at their boundaries

We further investigated the biological relevance of *hicream* clusters by focusing on the boundaries of their genomic projections in both datasets. First, using experimental ChIP-seq data, we observed a significant enrichment of active CTCF binding sites at cluster boundaries compared to their flanking regions, with an average of 21.1 peaks versus 16.8 in the neuronal differentiation study, and 5.7 peaks versus 3.8 in the CTCF depletion study (*P*-value $=2.52\times 10^{-16}$ and $6.26\times 10^{-8}$ respectively, Wilcoxon paired test; see also Fig. S8, available as supplementary data at *Bioinformatics* online for the neuronal differentiation study).

**Figure 5:**
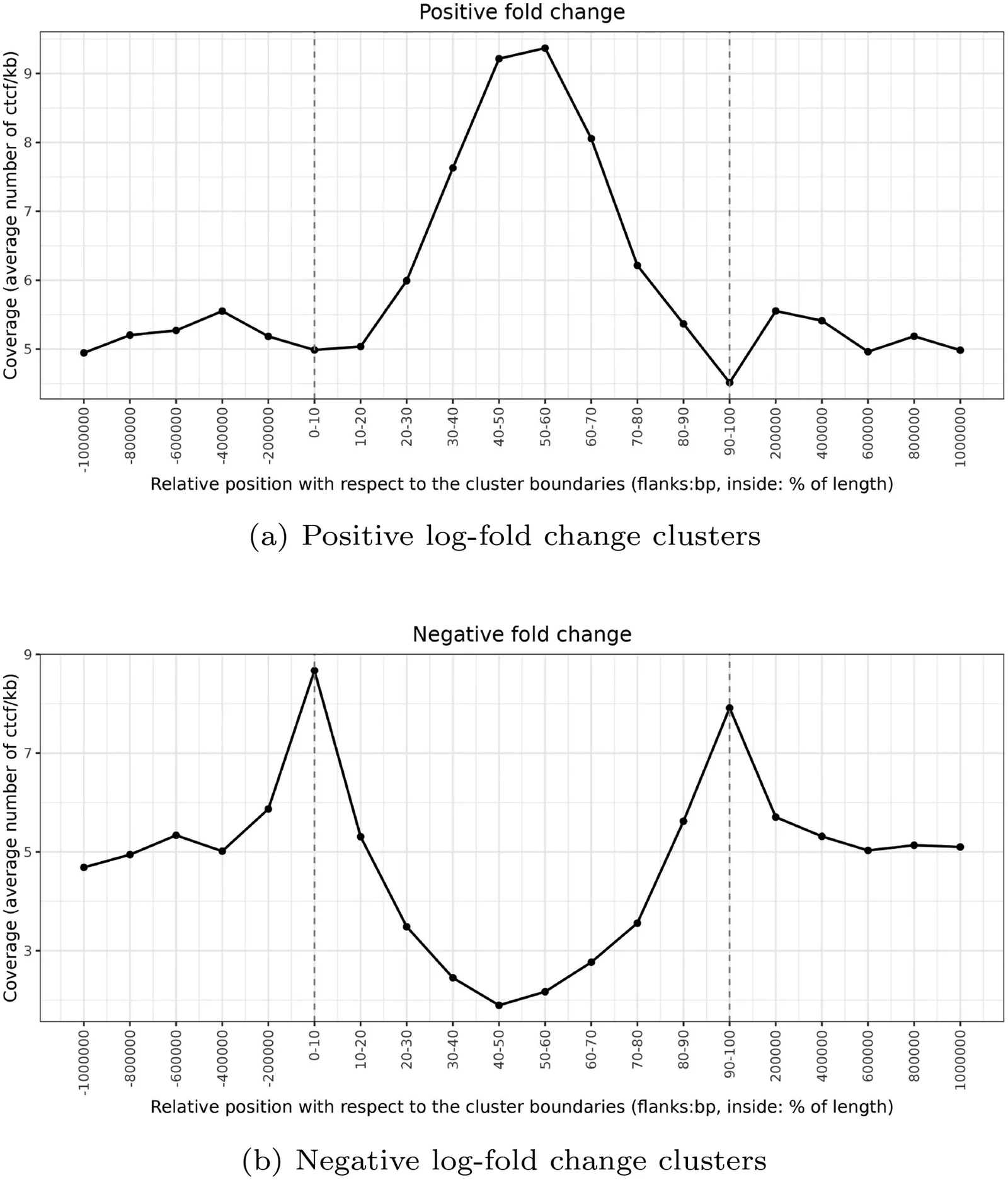
Distribution of CTCF peak coverage within and around the projected regions of hicream differential clusters at 200 kb resolution (CTCF depletion study). Positions on the *x*-axis correspond to left and right flanking regions of the projected boundaries and ten genomic intervals of size 10% of each cluster length between the two boundaries. Top (resp. Bottom): Clusters with a majority of positive (resp. negative) log-fold change pixels.

For the CTCF depletion study, we further examined the full CTCF occupancy profiles within and around the projected clusters depending on the sign of their logFC. As shown in Figure 5, the occupancy profiles differ strikingly between clusters with positive and negative logFC values, exhibiting complementary patterns. This difference is consistent with the known effects of CTCF depletion on topologically associating domains (TADs) (see Fig. S9 and Supplementary Section S3, available as supplementary data at *Bioinformatics* online). In brief, CTCF depletion is known to weaken TAD boundary insulation, allowing increased inter-TAD interactions across boundaries. As a result, pixels with increased interaction values after depletion are expected to localize above and around annotated TAD boundaries in Hi-C matrices, giving rise to clusters with positive logFC values and high CTCF binding site densities. Conversely, as CTCF depletion globally reduces TAD strength, intra-TAD interactions are expected to decrease. This effect generates differential clusters with negative logFC values and TAD-like CTCF profiles at their boundaries.

Last, we extended the enrichment analysis to all known transcription factor binding sites (TFBSs) from the JASPAR database (see Methods). For the neuronal differentiation study, CTCF showed the most significant enrichment (Supplementary File S2, available as supplementary data at *Bioinformatics* online). Functional analysis of all enriched transcription factors revealed an overrepresentation of Gene Ontology terms related to cell differentiation and neuronal processes, including regulation of neurogenesis, regulation of cell differentiation, brain development, axon development, and other neuronal functions (Table S1, available as supplementary data at *Bioinformatics* online).

For the CTCF depletion study, in contrast to the results obtained with the ChIP-seq data, no significantly enriched TFBS were identified. Since ChIP-seq data reflect active CTCF binding sites, whereas TFBS enrichment is based on computational predictions, this discrepancy may suggest that *hicream* more accurately identifies sites that are truly affected by the depletion experiment. Together, these results support the biological relevance of *hicream* differential clusters and demonstrate that *hicream* accurately captures spatial reorganization of chromatin interactions from real Hi-C data.

## 4 Discussion and conclusion

We have introduced *hicream*, a flexible three-step framework of Hi-C data differential analysis. On a semi-synthetic dataset with controlled ground truth, we compared both versions of *hicream* with the only other method that enables data-driven discovery of differential clusters in Hi-C data, *diffHic* clustering. Across all tested chromosomes and resolutions, both versions of *hicream* outperformed *diffHic clustering*. Fewer but larger differential clusters were obtained, highlighting its ability to capture coherent and interpretable differential genomic structures.

In real case studies, we further showed that *hicream* can detect differential clusters of arbitrary shape, including non-standard yet biologically relevant structures such as stripes. Moreover, the results were consistent with the distribution of active CTCF binding sites and TFBSs at cluster boundaries, further supporting the ability of *hicream* to accurately identify reorganized genomic regions.

Furthermore, the method is highly generic and modular: The method (and implementation) can accommodate alternative choices for differential analysis and clustering than those implemented in *hicream*. In particular, the differential analysis step can be extended to more complex experimental designs by incorporating covariates and testing specific hypotheses through flexible contrasts.

Moreover, because *post hoc* inference provides statistical guarantees independently of the clustering procedure, prior knowledge can also be used to define regions of interest. For example, candidate regions may be derived from structural predictions such as TADs, A/B compartments, or chromatin loops when targeting specific genomic structures. Prior knowledge could also be integrated into the clustering procedure itself, by constraining it to produce clusters of a specific shape (e.g. stripe-like). While the former is straightforward to implement in *hicream* as it only requires specifying candidate regions, the latter would entail designing new clustering procedures able to enforce structural constraints, which constitutes a stimulating methodological perspective for the present work.

Finally, the *post hoc* inference step currently relies on the Simes bound, which may be conservative. In settings with spatial dependence, a calibration strategy has been described to obtain sharper bounds (Blanchard *et al*. 2020), which is a challenging but interesting direction for future research.

## Supplementary Material

btag631_Supplementary_Data

## Data Availability

All datasets used in this article are available or described at https://doi.org/10.57745/IEUSLV. The scripts reproducing the results of this manuscript are all provided at https://forge.inrae.fr/scales/replication-repository-for-hicream-article.
