# Supplementary material for: hicream: A flexible framework to identify significantly different regions in Hi-C data
Source: Bioinformatics. 2026 Aug 25;42(9):btag631. doi: 10.1093/bioinformatics/btag631 (PMC13597124; doi:10.1093/bioinformatics/btag631)
Supplement: Supplementary file 1 [file btag631_supplementary_data.zip › jorge_etal_p2025-suppmat.pdf]

Supplementary information for "hicream: a flexible framework to identify significantly different regions in Hi-C data"

Elise Jorge, Toby Dylan Hocking, Pierre Neuvial, Nathalie Vialaneix, and Sylvain Foissac

Contents

S1 Supplementary methods 2
S1.1 Pixel-based differential analysis with **diffHic DA** . . . 2
S1.2 Connectivity constrained 2D hierarchical clustering . . . 2
S1.2.1 Variable transformation and normalization . . . 2
S1.2.2 Constraint to ensure unique connected component . . . 2
S1.2.3 Choice of 2D hierarchical clustering algorithm . . . 3

S2 Supplementary information on data processing 4
S2.1 Semi-synthetic dataset . . . 4
S2.2 CTCF depletion study . . . 4
S2.3 Neuronal differentiation study . . . 4
S2.4 Analysis of cluster shapes . . . 5
S2.5 Comparison with **TADCompare** and **dcHiC** results . . . 6
S2.6 CTCF enrichment . . . 6

S3 Expected impact of CTCF depletion 7

S4 Supplementary figures 8

S1 Supplementary methods

S1.1 Pixel-based differential analysis with diffHic DA

In **diffHic** differential analysis, a normalization step is performed using the correction of the MA plot with Locally Estimated Scatterplot Smoothing (LOESS) [Ballman et al., 2004] as implemented in the R package **csaw** [Lun and Smyth, 2016]. The statistical tests then rely on generalized linear models (GLM) based on a Negative Binomial (NB) distribution assumption for the pixel counts (as implemented in the R package **edgeR** [Robinson et al., 2010]). For each pixel (i, j) such that $i \leq j$, a log-linear model

$$\log \lambda_{ij}^{g,l} = \beta_{ij}^{(0)} + \beta_{ij}^{(1)} \mathbb{1}_{\{g=2\}} + \log N_{g,l},$$

is fitted, where for sample l in condition g, $\lambda_{ij}^{g,l} = \mathbb{E}(m_{ij}^{g,l})$ is the pixel-specific mean of the NB distribution, and $N_{g,l}$ is an estimate of the library size. The statistical test of the null hypothesis

$$\mathrm{H}_{0,ij}\colon \text{“}\beta_{ij}^{(1)} = 0\text{”}$$

for every pixel (i, j) results in a p-value half-matrix, $(\mathbf{P}_{ij})_{i,\,j=1\dots,p,\ i\leq j}$, which contains the result of $m \leq \frac{p(p+1)}{2}$ tested hypotheses, where m is the number of pixels for which at least one interaction has been observed in one of the r Hi-C matrices.

S1.2 Connectivity constrained 2D hierarchical clustering

S1.2.1 Variable transformation and normalization

For **hicream diff**, the clustering is performed using two variables: the adjusted p-value and log-fold change computed using **diffHic**. In practice, we first transform the variables into $(-\log_{10} \mathbf{P}^{\text{adj}}, \log_2 \text{FC})$ and then separately normalize them into $[0, 1]$ by applying a min-max normalization. The use of two log transformations with different bases (that correspond to the respective standard bases for these two quantities) is not an issue as the min-max normalization is invariant relative to log-base choice.

S1.2.2 Constraint to ensure unique connected component

The graph $\mathcal{G}$ links pixels that are adjacent in the Hi-C matrix in such a way that a pixel has, at most, 4 neighbors. However, due to missing bins in the data (*e.g*, pixels $q := (i, j)$ that have zero counts for a fixed q in all r matrices), some pixels could have no neighbors, potentially leading to a disconnected graph.

To solve this issue, we extend the definition of connectivity in order to minimize the number of connected components in the graph: $q_1 = (i_1, j_1)$ and $q_2 = (i_2, j_2)$ are connected in $\mathcal{G}$ iif one of the following condition is satisfied:

$$
\begin{aligned}
i_1 = i_2,\ j_2 &= \underset{j^\star > j_1,\,(i_2, j^\star)\in V}{\arg\min} |j^\star - j_1| \\
i_1 = i_2,\ j_2 &= \underset{j^\star < j_1,\,(i_2, j^\star)\in V}{\arg\min} |j^\star - j_1| \\
j_1 = j_2,\ i_2 &= \underset{i^\star > i_1,\,(i^\star, j_2)\in V}{\arg\min} |i^\star - i_1| \\
j_1 = j_2,\ i_2 &= \underset{i^\star < i_1,\,(i^\star, j_2)\in V}{\arg\min} |i^\star - i_1|
\end{aligned}
$$

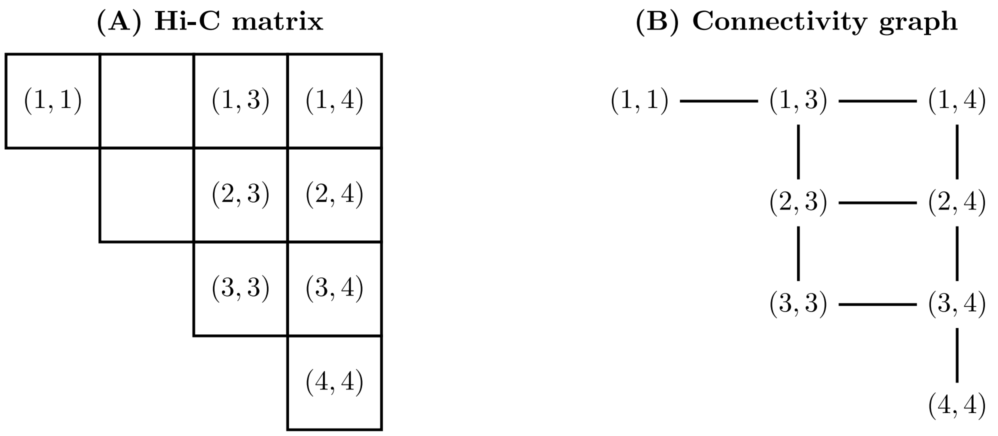

Figure S1: **Illustration of the connectivity constraint.** (A) Extract of the right upper part of a Hi-C matrix where non observed pixels are identified by empty squares (the other pixels are observed). (B) Resulting connectivity graph used in our 2D clustering.

Figure S1 gives an example of the resulting graph: Pixel $(1,1)$ has no direct contiguous neighbor as the bin 2 is "missing", but, the latter definition ensures that Pixel $(1,1)$ is connected to the rest of the graph through its closest right pixel, *i.e*, Pixel $(1,3)$. In practice, this results in a graph that contains only one connected component. However, in some rare cases (e.g., if for a pixel (i,j), there exists no other pixel of the form (i',j) or (i,j')), the graph could contain more than one connected component.

S1.2.3 Choice of 2D hierarchical clustering algorithm

We propose to perform a two-dimensional connectivity-constrained agglomerative clustering. In short, the agglomerative clustering is based on Ward's clustering method [Ward Jr, 1963] and we used the constrained version formally defined in Lebart [1978], Grimm [1987], Gordon [1996]. Here, the constraint is encoded in graph $\mathcal{G}$. This approach was already used by Thirion et al. [2014] to obtain clustering of fMRI pixels arranged in a 3D space. This results in a dendrogram, which can contain a few rever-

sals (due to the contiguity constraint; see Randriamihamison et al. [2021]), from which we can obtain clusters $\{C_1, \cdots, C_K\}$ that form a partition of the pixels by selecting a level at which to cut the hierarchy.

S2 Supplementary information on data processing

S2.1 Semi-synthetic dataset

Semi-synthetic data were obtained using an ENCODE dataset [The ENCODE Project Consortium, 2012]. More precisely, we used the Hi-C experiment performed on a human colon sample (accession number: ENCSR295BDK). The raw sequencing reads were processed using nf-core/hic pipeline [Ewels et al., 2020] v1.2.2 on the assembly version GRCh38 of the human genome in order to obtain Hi-C matrices. Processed Hi-C matrices thus correspond to five technical replicates for three different chromosomes at three different resolutions and are available at `https://doi.org/10.57745/LR0W9R`.

S2.2 CTCF depletion study

We used a large scale dataset from an existing study on the role of CTCF during cell cycle progression in post-mitotic G1E-ER4 mouse cells [Zhang et al., 2021]. The study was conducted under two conditions: either accute depletion of CTCF through an auxin-inducible degron system (CTCF- condition, with three replicates) or in the control condition without auxin-induced depletion (CTCF+ condition, with three replicates). Raw interaction counts of the six Hi-C matrices were directly downloaded from the GEO platform using the accession number GSE168251 from `https://www.ncbi.nlm.nih.gov/geo/query/acc.cgi?acc=GSE168251` (`GSM5133388_120min_no_a_rep1.cool.gz`, `GSM5133389_120min_no_a_rep2.cool.gz`, `GSM5133390_120min_no_a_rep3.cool.gz`, `GSM5133391_120min_with_a_rep1.cool.gz`, `GSM5133392_120min_with_a_rep2.cool.gz`, and `GSM5133393_120min_with_a_rep3.cool.gz`). Matrices in cool format were exported in HiC-Pro text format by chromosome at 10 kb resolution and coarsened to 200 kb resolution using Bioconductor package **HiContacts**. Resulting files by replicate and chromosome along with the genome-wide index are provided at `https://doi.org/10.57745/IEUSLV`. Corresponding narrow peaks from CTCF ChIP-seq experiments were downloaded from GEO (accession GSE129997). This data corresponds to the CTCF+ condition, *e.g.*, to the expected positions of CTCF sites in a control condition [Zhang et al., 2019].

S2.3 Neuronal differentiation study

We used a large scale dataset from an existing study on neuronal differentiation in mouse [Bonev et al., 2017] and processed the data as described in Maigné et al. [2025]. In brief, sequencing reads were downloaded from the SRA (`https://www.ncbi.nlm.nih.gov/sra`) using the accession ID SRP101791 (GEO: GSE96107) and processed using the nf-core/hic pipeline v1.3.0 on

the assembly version GRCm39 of the mouse genome using the following arguments: `--min_mapq 10 --digestion 'dpnii' --min_insert_size 20 --max_insert_size 1000 --bwt2_opts_end2end '--very-sensitive -L 20 --score-min L,-0.6,-0.3 --end-to-end --reorder' --bwt2_opts_trimmed '-5 5 --very-sensitive -L 20 --score-min L,-0.6,-0.3 --end-to-end --reorder'`. Samples correspond to 2 cellular differentiation stages during neuronal development: mouse embryonic stem cells (ES) and cortical neurons (CN), with 4 replicates per cell type (GEO Accession IDs: GSM2533818, GSM2533819, GSM2533820, and GSM2533821 for ES, and GSM2533826, GSM2533827, GSM2533828, and GSM2533829 for CN). Corresponding ChIP-seq data were obtained from the ReMap ChIP-seq track of the UCSC Table Browser, selecting the CTCF.mESC lines of the ReMapTFs Table.

Processed data are available at `https://doi.org/10.57745/IEUSLV`.

S2.4 Analysis of cluster shapes

Both datasets from the CTCF depletion and the neuronal differentiation studies were analyzed with **hicream** using the **hicream diff** clustering mode.

To characterize the shape of the resulting clusters, three shape-related metrics were computed and assigned to each cluster: a triangle-, a stripe-, and a square-score.

- `triangle_score`: defined as $s/(b^2/2)$, where s is the cluster size (number of pixels) and b the total number of distinct genomic bins involved in the cluster, computed as the union of all row and column bin indices across its contained pixels. This score reaches its maximum when the cluster resembles a topologically associating domain (TAD), i.e. a triangular structure along the diagonal of the contact matrix.

- `stripe_score`: defined as $\log(p_i/p_j)$, where $p_i = i_{\max} - i_{\min} + 1$ and $p_j = j_{\max} - j_{\min} + 1$ are the genomic projections of the cluster along its row and column axes, respectively. A value of 0 is expected for radially symmetric clusters, whereas deviations from 0 indicate elongation along one axis. The sign encodes the orientation of the elongation, while the absolute value is used for the subsequent PCA.

- `square_score`: defined as s/P, where s is the number of pixels in the cluster and P its perimeter, i.e. the total number of pixel sides not shared with any neighboring pixel belonging to the same cluster. Given that each pixel occupies a unit square, this score is maximized when the cluster approximates a square geometry.

These three shape-related metrics, along with the TDP and the absolute log-fold change (logFC) were then used to perform a Principal Component Analysis (PCA) on the set of differential clusters for each study.

S2.5 Comparison with TADCompare and dcHiC results

hicream results obtained for the neuronal differentiation study were compared with **TADCompare** and **dcHiC** results.

TADCompare was run on all chromosomes of the Hi-C data from the neuronal differentiation study. Since the method does not allow for replicates, the replicates were previously merged by summing the Hi-C matrices from the same condition. Genomic regions corresponding to significantly shifted TADs enriched in CN (resp. ES) counts were then extracted.

Selected clusters were first projected onto the genome to identify the corresponding genomic boundaries and their flanking bins. The boundaries were defined as the first and last genomic bins among all interacting bins within each cluster. We then tested for the enrichment in bins identified as "significantly shifted" by **TADCompare** at the boundaries compared with the flanking bins. Specifically, we computed the number of such bins falling in the boundary bins versus those falling in the flanking bins and we compared the resulting distributions using a paired Wilcoxon test.

dcHiC was run on all chromosomes of the Hi-C data from the neuronal differentiation study. Pixels (i, j) from every chromosome were then described with:

- the TDP of the cluster they belong to;
- the type of compartments the two corresponding bins belong to in both conditions, as obtained from **dcHiC**. We encoded this information using the notation $\mathrm{X}_i^{\mathrm{ES}}\mathrm{X}_j^{\mathrm{ES}}2\mathrm{X}_i^{\mathrm{CN}}\mathrm{X}_j^{\mathrm{CN}}$ where $X_\ell^k \in \{\mathrm{A, B}\}$ is the compartment of bin ℓ (i or j) in condition k (ES or CN). For instance, "AA2BB" describes a pixel between two bins in "A" compartment in ES condition that becomes a pixel between two bins in "B" compartment in CN.

Distribution of positive TDP with respect to pixel compartment types were then obtained, considering two groups of compartment types: the group characterizing compartment changes being composed of types "AA2BB" and "BB2AA" and the other group being composed of all the other cases. In addition, a Wilcoxon test was performed to compare TDP distributions between the two groups of compartment types.

S2.6 CTCF enrichment

Results obtained on both the CTCF depletion and the neuronal differentiation datasets were compared with CTCF signals. Selected **hicream** clusters were first projected onto the genome to identify the corresponding genomic boundaries and their flanking bins. The boundaries were defined as the first and last genomic bins among all interacting bins within each cluster. We then tested for CTCF enrichment at the boundaries compared with the flanking bins, using the genomic coordinates of active CTCF binding sites identified by ChIP-seq experiments. Specifically, we computed the number of CTCF peaks overlapping the boundary bins versus the flanking bins and compared the resulting distributions using a paired Wilcoxon test.

In addition, the distribution of active CTCF binding sites was further characterized by profiling their relative positions with respect to the cluster boundaries. To do this, for each selected cluster $C \subset \{(i, j) | 1 \leq i \leq j \leq p\}$, we identified the two boundaries of its projection on the chromosome, which correspond to the bins $\underline{i}$ and $\overline{j}$ for which we have:

$$\begin{aligned}
&\underline{i} \leq \overline{j} \\
&\exists j^* \text{ s.t, } (\underline{i}, j^*) \in C \text{ and } \forall (i, j) \in C,\ \underline{i} \leq i \\
&\exists i^* \text{ s.t, } (i^*, \overline{j}) \in C \text{ and } \forall (i, j) \in C,\ \overline{j} \geq j.
\end{aligned}$$

Then, the average number of CTCF peaks per kb was computed for 20 genomic intervals:

- 10 genomic intervals of equal sizes that each correspond to 10% of the total length of the genomic interval $[\underline{i}, \overline{j}]$;
- 5 flanking regions of equal sizes at 1Mb left (resp. right) of boundary $\underline{i}$ (resp. $\overline{j}$).

Moreover, we tested all cluster boundaries for statistical enrichment of known transcription factor binding sites (TFBSs) from the JASPAR database (CORE Vertebrates 2024) using the AME tool from the MEME Suite (v5.5.4). The background model for this enrichment analysis was built by extracting the flanking sequences around each cluster in order to account for local biases in base composition.

Last, genes with enriched binding motifs from the TFBS analysis were also subjected to a Gene Ontology functional analysis using the Panther Classification System tool from `https://geneontology.org` (PANTHER GO-Slim Biological Process, Fisher exact test and Bonferroni multiple testing correction).

S3 Expected impact of CTCF depletion

Figure S9 illustrates the expected impact of CTCF depletion on the data, particularly at the TAD level. As CTCF is widely known to be associated with TAD boundary formation, we expect a disruption of these structures in the CTCF- condition. Therefore, for two TADs separated by a boundary in the CTCF+ condition, we expect this boundary to be weakened or deleted in the CTCF- condition. This allows more frequent interactions between two positions located in contiguous TADs and leads to differential clusters of positive log-fold change located within inter-TAD regions. In parallel, we expect less frequent interactions within TADs and thus, intra-TAD differential clusters of negative log-fold change.

In conclusion, after projecting each cluster and representing the average CTCF coverage as described in Section S2.6, two different behaviors are expected whether the clusters have an average log-fold change that is positive or negative. If the average log-fold change is negative, we expect a bi-modal distribution with two peaks in CTCF coverage corresponding to the boundary regions. On the other hand, if the average log-fold change is positive, we expect a unimodal distribution with a peak of CTCF coverage located between the two boundaries.

S4 Supplementary figures

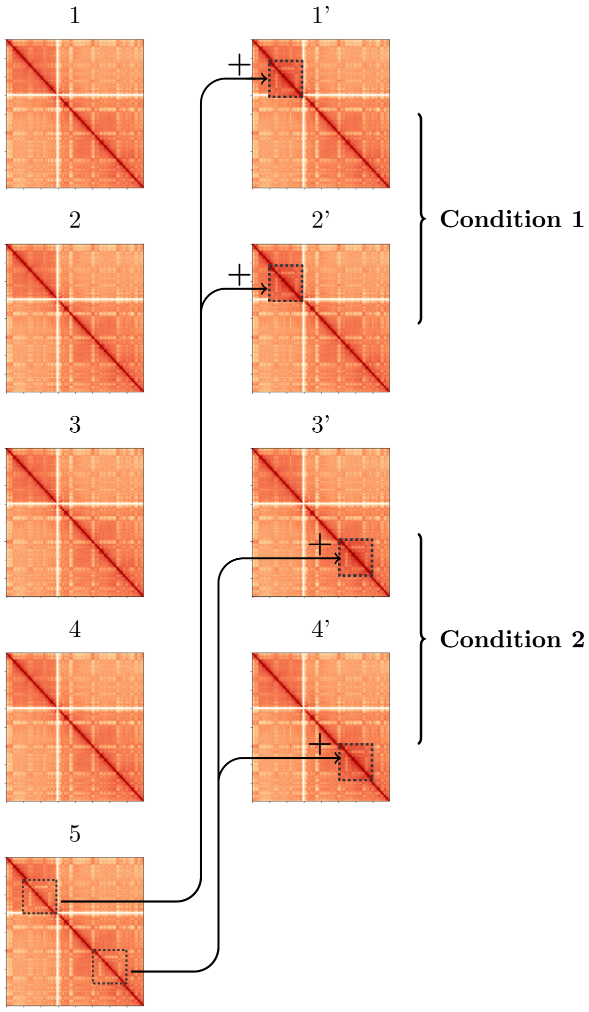

Figure S2: **Semi-synthetic setting.** The original Hi-C matrices consist of five technical replicates from the ENCODE project (left). An artificial Condition 1 was arbitrarily created using Hi-C matrices 1 and 2, to which counts from the fifth matrix were locally added within a predefined target region. This region corresponds to pixels whose interacting bins are located between the 20th and 40th quantiles of chromosome positions. Condition 2 was built using matrices 3 and 4, with counts from a different region of the fifth matrix added. This second region corresponds to pixels whose interacting bins are located between the 60th and 80th quantiles of chromosome positions.

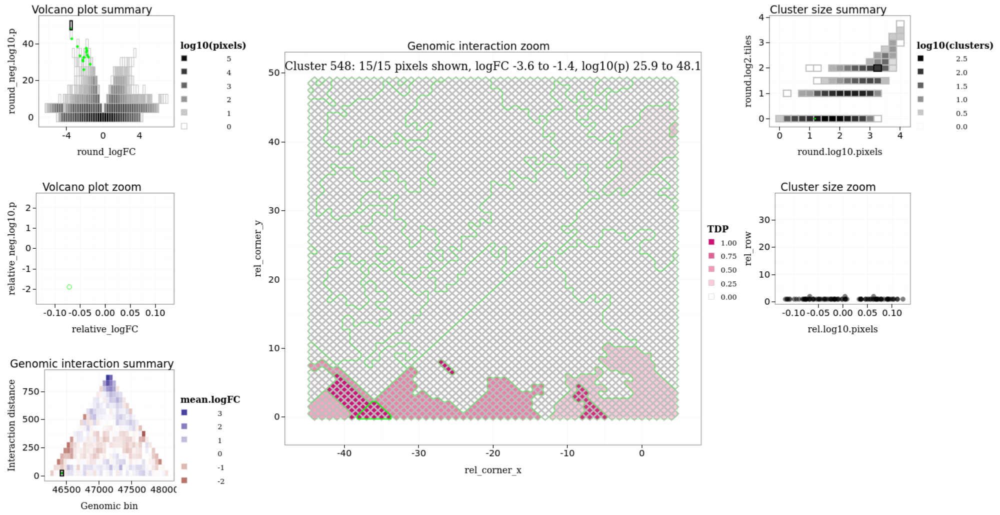

Figure S3: **Neuronal differentiation study: Dynamic visualization of hicream results for chromosome 18 at 50kb resolution with animint2.** The *tile* (genomic region of size 50×50 bins) selected on the *Genomic interaction summary* frame is displayed on the *Genomic interaction zoom* panel. Clusters are outlined in green and each pixel is colored relative to the TDP (True Discovery Proportion) computed using **hicream** for its cluster. Each pixel of the cluster selected on the *Genomic interaction zoom* panel is displayed as a green point on the $\log \mathrm{FC}/\ p$-value volcano plot of *Volcano plot summary*. Clusters can be selected based on the *Volcano plot summary* and *Volcano plot zoom*. It is also possible to select clusters based on their size through the *Cluster size summary* and *Cluster size zoom* panels. Selection of a cluster or tile in one frame automatically updates the other frames.

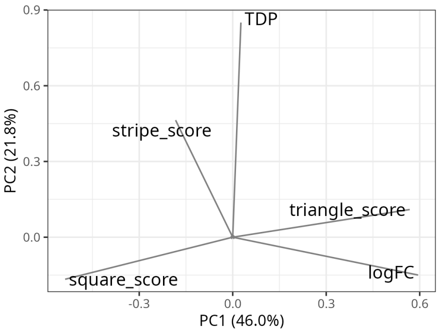

(a) CTCF depletion study

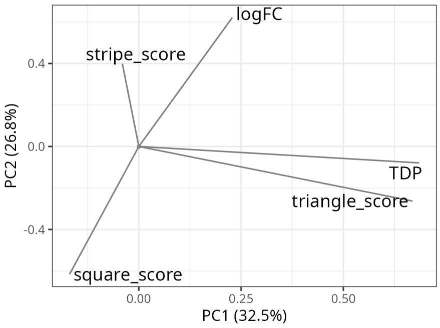

(b) Neuronal differentiation study

Figure S4: **PCA variable loadings in the CTCF depletion (a) and the neuronal differentiation (b) study.** Principal Component Analysis was perfomed on the differential clusters using their functional (TDP, |logFC|) and shape-related (`triangle_score`, |`stripe_score`|, `square_score`) features. Results show a different association of TDP with the shape-related features: TDP is more associated with `stripe_score` in the CTCF depletion study and with `triangle_score` in the neuronal differentiation study.

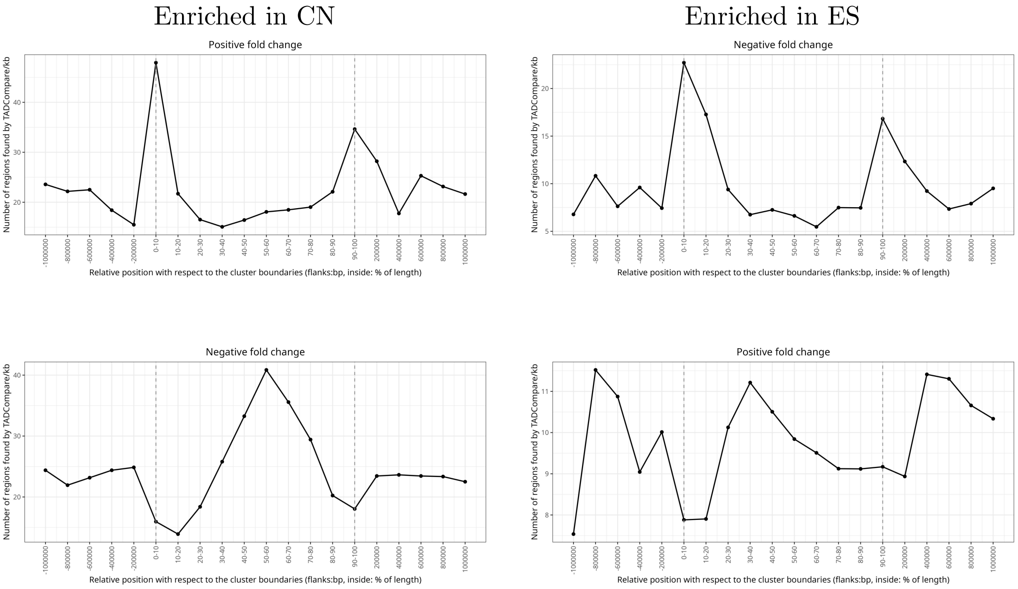

Figure S5: **Comparison between hicream and TADCompare results (neuronal differentiation study).** Enrichment of differentially shifted positions found by **TADCompare** at **hicream** cluster boundaries. Left: Regions enriched in CN for **TADCompare**. Right: Regions enriched in ES for **TADCompare**. First row: **hicream** cluster with logFC sign consistent with **TADCompare** enrichment (*e.g.* positive for CN enrichment and negative for ES enrichment). Second row: **hicream** cluster with logFC sign opposite to **TADCompare** enrichment.

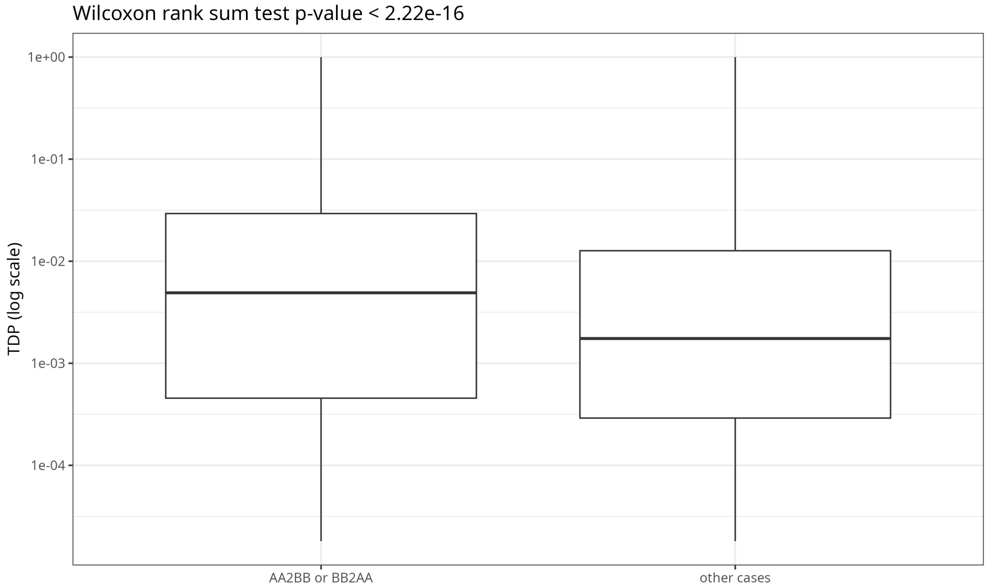

Figure S6: **Comparison between hicream and dcHiC results (neuronal differentiation study).** Distribution (at pixel level) of the pixel cluster TDP with respect to its **dcHiC** status: major compartment change (AA2BB or BB2AA) or other cases. Only pixels in clusters with a positive TDP were considered for this analysis.

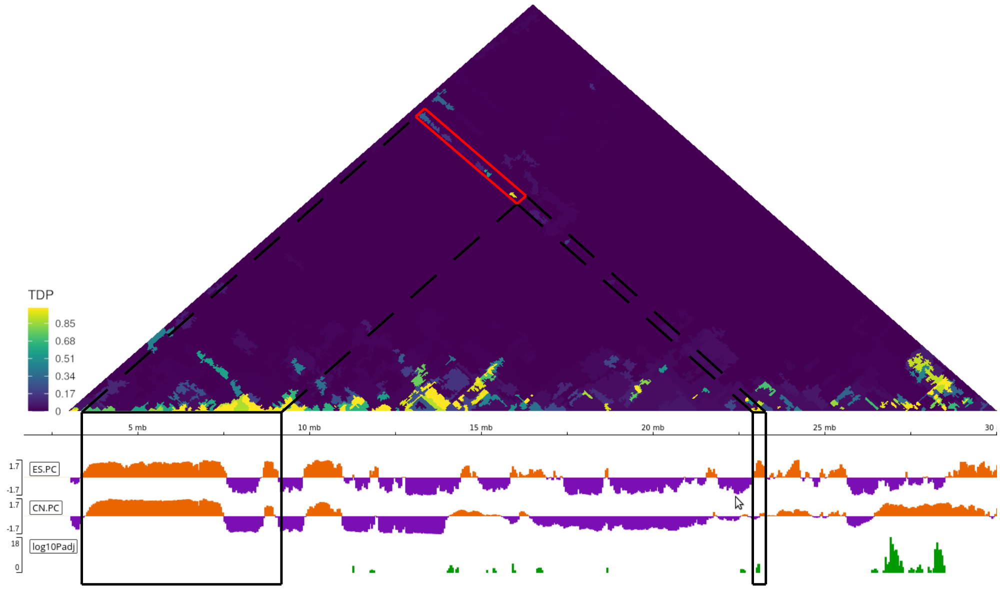

Figure S7: **Neuronal differentiation study: hicream and dcHiC results for specific section of chromosome 19.** (Top) **hicream** results for a specific part of chromosome 19 starting after 0Mb and ending at 30Mb. (Bottom) **dcHiC** results for the same chromosome section, displaying compartment predicitons in ES and CN conditions as well as the (log10-adjusted) p-value for each bin. Orange values correspond to A compartment while purple values correspond to B compartment. A group of 7 selected clusters is outlined in red. The two specific parts of **dcHiC** results corresponding to the selected cluster projections are outlined in black.

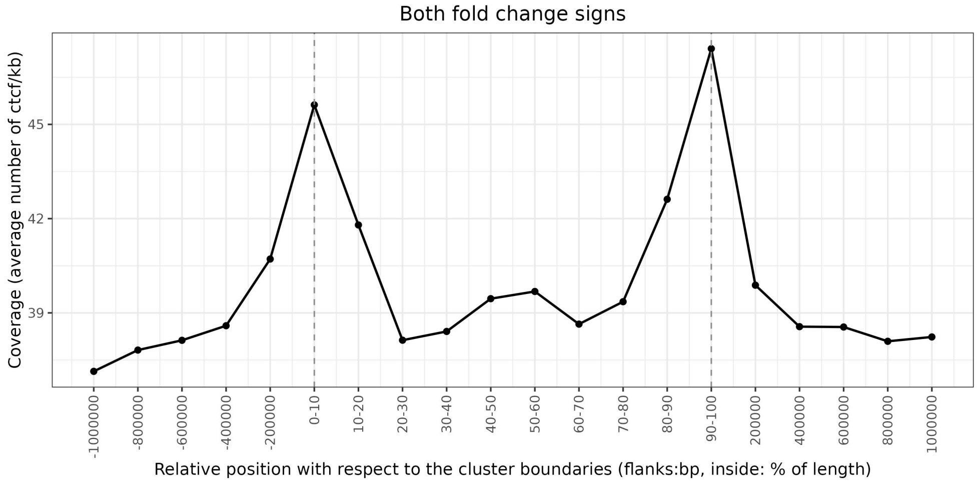

Figure S8: **Neuronal differentiation study: Average distribution of CTCF coverage in boundary regions of differential clusters across all chromosomes at 50kb resolution.** Positions on the x-axis correspond to left and right flanking regions of projected boundaries (each corresponding to five bins) and ten genomic subintervals of size 10% of the length between the two cluster boundaries.

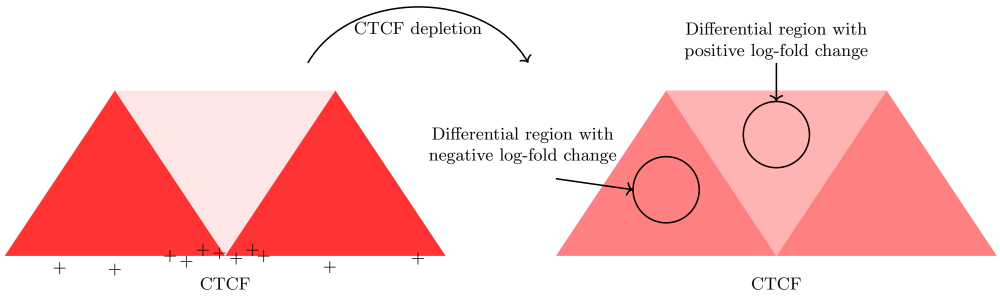

Figure S9: **Illustration of CTCF depletion effect.** On the left, two TAD-like regions are separated by a boundary. The CTCF density is high around that boundary but low within the two flanking regions. On the right, the induced CTCF depletion lowers the global density of CTCF and primarily impacts the boundary, allowing more frequent interactions between positions on each side of the boundary. This creates two types of differential regions with positive and negative log-fold change.

References

K. V. Ballman, D. E. Grill, A. L. Oberg, and T. M. Therneau. Faster cyclic loess: normalizing RNA arrays via linear models. *Bioinformatics*, 20(16):2778–2786, 2004. doi: 10.1093/bioinformatics/bth327.

B. Bonev, N. M. Cohen, Q. Szabo, L. Fritsch, G. L. Papadopoulos, Y. Lubling, X. Xu, X. Lv, J.-P. Hugnot, A. Tanay, and G. Cavalli. Multiscale 3D genome rewiring during mouse neural development. *Cell*, 171(3):557–572.e24, 2017. doi: 10.1016/j.cell.2017.09.043.

P. A. Ewels, A. Peltzer, S. Fillinger, H. Patel, J. Alneberg, A. Wilm, M. U. Garcia, P. Di Tommaso, and S. Nahnsen. The nf-core framework for community-curated bioinformatics pipelines. *Nature Biotechnology*, 38:276–278, 2020. doi: 10.1038/s41587-020-0439-x.

A. Gordon. A survey of constrained classification. *Computational Statistics & Data Analysis*, 21(1):17–29, 1996. doi: 10.1016/0167-9473(95)00005-4.

E. C. Grimm. CONISS: a FORTRAN 77 program for stratigraphically constrained analysis by the method of incremental sum of squares. *Computers & Geosciences*, 13(1):13–35, 1987. doi: 10.1016/0098-3004(87)90022-7.

L. Lebart. Programme d'agrégation avec contraintes. *Les Cahiers de l'Analyse des Données*, 3(3):275–287, 1978. URL `http://www.numdam.org/item?id=CAD_1978__3_3_275_0`.

A. T. Lun and G. K. Smyth. csaw: a Bioconductor package for differential binding analysis of ChIP-seq data using sliding windows. *Nucleic Acids Research*, 44(5):e45, 2016. doi: 10.1093/nar/gkv1191.

E. Maigné, C. Kurylo, M. Zytnicki, and S. Foissac. HiCDOC: chromatin compartment prediction and differential analysis from Hi-C data with replicates. bioRxiv preprint. DOI: 10.1101/2025.09.18.677058, 2025.

N. Randriamihamison, N. Vialaneix, and P. Neuvial. Applicability and interpretability of Ward's hierarchical agglomerative clustering with or without contiguity constraints. *Journal of Classification*, 38:363–389, 2021. doi: 10.1007/s00357-020-09377-y.

M. D. Robinson, D. J. McCarthy, and G. K. Smyth. edger: a bioconductor package for differential expression analysis of digital gene expression data. *bioinformatics*, 26(1):139–140, 2010. doi: 10.1093/bioinformatics/btp616.

The ENCODE Project Consortium. An integrated encyclopedia of DNA elements in the human genome. *Nature*, 489(7414):57–74, 2012. doi: 10.1038/nature11247.

B. Thirion, G. Varoquaux, E. Dohmatob, and J.-B. Poline. Which fMRI clustering gives good brain parcellations? *Frontiers in Neuroscience*, 8:167, 2014. doi: 10.3389/fnins.2014.00167.

J. H. Ward Jr. Hierarchical grouping to optimize an objective function. *Journal of the American statistical association*, 58(301):236–244, 1963.

H. Zhang, D. J. Emerson, T. G. Gilgenast, K. R. Titus, Y. Lan, P. Huang, D. Zhang, H. Wang, C. A. Keller, B. Giardine, R. C. Hardison, J. E. Phillips-Cremins, and G. A. Blobel. Chromatin structure dynamics during the mitosis-to-G1 phase transition. *Nature*, 576(7785):158–162, 2019. doi: 10.1038/s41586-019-1778-y.

H. Zhang, J. Lam, D. Zhang, Y. Lan, M. W. Vermunt, C. A. Keller, B. Giardine, R. C. Hardison, and G. A. Blobel. CTCF and transcription influence chromatin structure re-configuration after mitosis. *Nature Communications*, 12:5157, 2021. doi: 10.1038/s41467-021-25418-5.
