# Supplementary material for: hicream: A flexible framework to identify significantly different regions in Hi-C data
Source: Bioinformatics. 2026 Aug 25;42(9):btag631. doi: 10.1093/bioinformatics/btag631 (PMC13597124; doi:10.1093/bioinformatics/btag631)
Supplement: Supplementary file 1 [file btag631_supplementary_data.zip › SuppFileS1.pdf]

AME
Analysis of Motif Enrichment

For further information on how to interpret these results please access http://meme-suite.org/doc/ame-output-format.html.
To get a copy of the MEME software please access https://meme-suite.org.

If you use AME in your research, please cite the following paper:
Robert C. McLeay and Timothy L. Bailey, "Motif Enrichment Analysis: a unified framework and an evaluation on ChIP data", *BMC Bioinformatics*, **11**:165, 2010. [full text]

Enriched Motifs | Input Files | Program information | Results in TSV Format ? | Positive sequences for each motif ?

Enriched Motifs

Sequence motif score: avg_odds
Background model source: letter frequencies in (primary) sequences file (res/ctcf/200000/clusterboundaries_mintpr0.1_200000_allfc_firstlastbins.fasta)
Background model frequencies: 0.285516,0.214484,0.214484,0.285516
Total pseudocount added to a motif column: 0.1

Statistical test: Fisher's exact test (optimized over motif scores)
Labeling positives: all 2406 primary sequences are labeled as 'positive'; all 2394 control sequences are labeled as 'negative'
Classifying positives: sequences with the motif scores ≥ 'TP Thresh' are classified as positive

Logo	Database ?	ID ?	Alt ID ?	*p*-value ?	*E*-value ?	Pos Thresh ?	Pos ?	Neg ?	TP Thresh ?	TP (%) ?	FP (%) ?	Pearsons's CC (ρ) ?	Spearman Rank CC (ρ) ?

No motifs passed the *E*-value threshold (10).

Input Files

Alphabet

Background source: built from the (primary) sequences

Name ?	Bg. ?				Bg. ?	Name ?
Adenine	0.285516	A	~	T	0.285516	Thymine
Cytosine	0.214484	C	~	G	0.214484	Guanine

Sequences

Primary Sequences	Number	Control Sequences
res/ctcf/200000/clusterboundaries_mintpr0.1_200000_allfc_firstlastbins.fasta	2406	res/ctcf/200000/clusterboundaries_mintpr0.1_200000_allfc_fla

Motifs

Database	Source	Motif Count
JASPAR2024 CORE vertebrates non-redundant pfms meme	data/motif_databases/JASPAR/2024/JASPAR2024_CORE_vertebrates_non-redundant_pfms_meme.txt	879

AME version
5.5.4 (Release date: Fri Jun 16 12:19:08 2023 -0700)
Copyright © Robert McLeay & Timothy Bailey meme-suite@uw.edu, 2009.

Reference
Robert C. McLeay and Timothy L. Bailey, "Motif Enrichment Analysis: a unified framework and an evaluation on ChIP data", *BMC Bioinformatics*, **11**:165, 2010. [full text]

Command line
```
ame --oc res/ctcf/200000//flankingbackground_200000 --control
res/ctcf/200000/clusterboundaries_mintpr0.1_200000_allfc_flanks.fasta
res/ctcf/200000/clusterboundaries_mintpr0.1_200000_allfc_firstlastbins.fasta
data/motif_databases/JASPAR/2024/JASPAR2024_CORE_vertebrates_non-redundant_pfms_meme.txt
```
