# Supplementary material for: hicream: A flexible framework to identify significantly different regions in Hi-C data
Source: Bioinformatics. 2026 Aug 25;42(9):btag631. doi: 10.1093/bioinformatics/btag631 (PMC13597124; doi:10.1093/bioinformatics/btag631)
Supplement: Supplementary file 1 [file btag631_supplementary_data.zip › SuppFileS2.pdf]

AME
Analysis of Motif Enrichment

For further information on how to interpret these results please access http://meme-suite.org/doc/ame-output-format.html.
To get a copy of the MEME software please access https://meme-suite.org.

If you use AME in your research, please cite the following paper:
Robert C. McLeay and Timothy L. Bailey, "Motif Enrichment Analysis: a unified framework and an evaluation on ChIP data", *BMC Bioinformatics*, **11**:165, 2010. [full text]

Enriched Motifs | Input Files | Program information | Results in TSV Format ? | Positive sequences for each motif ?

Enriched Motifs

Sequence motif score: avg_odds
Background model source: letter frequencies in (primary) sequences file (res/bonev/50000/clusterboundaries_mintpr0.1_50000_allfc_firstlastbins.fasta)
Background model frequencies: 0.284679,0.215321,0.215321,0.284679
Total pseudocount added to a motif column: 0.1

Statistical test: Fisher's exact test (optimized over motif scores)
Labeling positives: all 8502 primary sequences are labeled as 'positive'; all 8502 control sequences are labeled as 'negative'
Classifying positives: sequences with the motif scores ≥ 'TP Thresh' are classified as positive

E-value threshold for reporting results: 10

Logo	Database ?	ID ?	Alt ID ?	*p*-value ?	*E*-value ?	TP Thresh ?
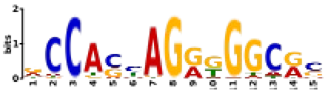	JASPAR2024 CORE vertebrates non-redundant pfms meme	MA0139.2	CTCF	8.87e-13	7.80e-10	4.43
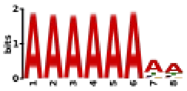	JASPAR2024 CORE vertebrates non-redundant pfms meme	MA1125.2	ZNF384	5.64e-7	4.96e-4	1.00
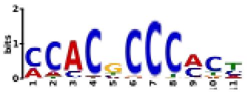	JASPAR2024 CORE vertebrates non-redundant pfms meme	MA0747.2	SP8	3.08e-6	2.71e-3	1.10
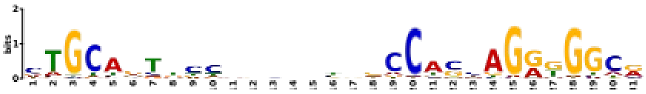	JASPAR2024 CORE vertebrates non-redundant pfms meme	MA1929.2	CTCF	6.99e-6	6.14e-3	29.06
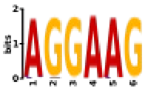	JASPAR2024 CORE vertebrates non-redundant pfms meme	MA2326.1	IKZF2	7.02e-6	6.17e-3	1.01
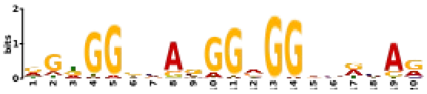	JASPAR2024 CORE vertebrates non-redundant pfms meme	MA1723.2	PRDM9	7.75e-6	6.82e-3	1.04
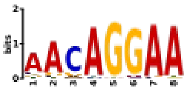	JASPAR2024 CORE vertebrates non-redundant pfms meme	MA1508.2	IKZF1	1.92e-5	1.69e-2	1.02
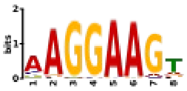	JASPAR2024 CORE vertebrates non-redundant pfms meme	MA0136.4	Elf5	3.41e-5	2.99e-2	1.00
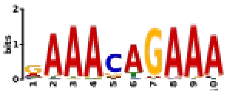	JASPAR2024 CORE vertebrates non-redundant pfms meme	MA1623.2	Stat2	6.75e-5	5.93e-2	1.01
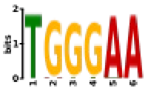	JASPAR2024 CORE vertebrates	MA1116.2	RBPJ	1.12e-	9.81e-	1.00

	non-redundant pfms meme			4	2	
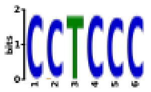	JASPAR2024 CORE vertebrates non-redundant pfms meme	MA1965.2	SP5	1.89e-4	1.66e-1	1.17
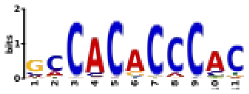	JASPAR2024 CORE vertebrates non-redundant pfms meme	MA1107.3	KLF9	4.60e-4	4.04e-1	1.03
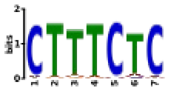	JASPAR2024 CORE vertebrates non-redundant pfms meme	MA0508.4	PRDM1	1.12e-3	9.88e-1	1.01
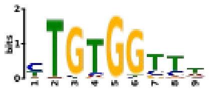	JASPAR2024 CORE vertebrates non-redundant pfms meme	MA0002.3	Runx1	1.31e-3	1.15e0	1.00
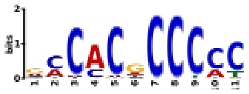	JASPAR2024 CORE vertebrates non-redundant pfms meme	MA0741.1	KLF16	2.07e-3	1.82e0	1.21
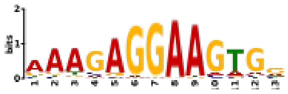	JASPAR2024 CORE vertebrates non-redundant pfms meme	MA0080.7	Spi1	3.59e-3	3.16e0	1.01
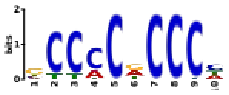	JASPAR2024 CORE vertebrates non-redundant pfms meme	MA0599.1	KLF5	4.22e-3	3.71e0	1.18
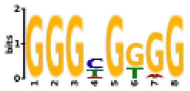	JASPAR2024 CORE vertebrates non-redundant pfms meme	MA0493.3	KLF1	5.00e-3	4.39e0	1.04
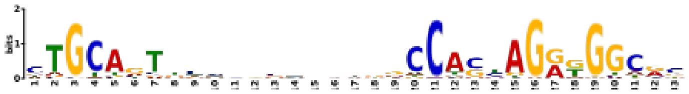	JASPAR2024 CORE vertebrates non-redundant pfms meme	MA1930.2	CTCF	6.28e-3	5.52e0	12.15

Input Files

Alphabet

Background source: built from the (primary) sequences

Name	Bg.				Bg.	Name
Adenine	0.284679	A	~	T	0.284679	Thymine
Cytosine	0.215321	C	~	G	0.215321	Guanine

Sequences

Primary Sequences	Number	Control Sequences
res/bonev/50000/clusterboundaries_mintpr0.1_50000_allfc_firstlastbins.fasta	8502	res/bonev/50000/clusterboundaries_mintpr0.1_50000_allfc_fla

Motifs

Database	Source	Motif Count
JASPAR2024 CORE vertebrates non-redundant pfms meme	data/motif_databases/JASPAR/2024/JASPAR2024_CORE_vertebrates_non-redundant_pfms_meme.txt	879

AME version

5.5.4 (Release date: Fri Jun 16 12:19:08 2023 -0700)
Copyright © Robert McLeay & Timothy Bailey meme-suite@uw.edu, 2009.

Reference

Robert C. McLeay and Timothy L. Bailey, "Motif Enrichment Analysis: a unified framework and an evaluation on ChIP data", *BMC Bioinformatics*, **11**:165, 2010. [full text]

Command line

```
ame --oc res/bonev/50000//flankingbackground_50000 --control
res/bonev/50000/clusterboundaries_mintpr0.1_50000_allfc_flanks.fasta
res/bonev/50000/clusterboundaries_mintpr0.1_50000_allfc_firstlastbins.fasta
data/motif_databases/JASPAR/2024/JASPAR2024_CORE_vertebrates_non-redundant_pfms_meme.txt
```
