# Supplementary material for: hicream: A flexible framework to identify significantly different regions in Hi-C data
Source: Bioinformatics. 2026 Aug 25;42(9):btag631. doi: 10.1093/bioinformatics/btag631 (PMC13597124; doi:10.1093/bioinformatics/btag631)
Supplement: Supplementary file 1 [file btag631_supplementary_data.zip › TableS1.pdf]

Analysis Type:	PANTHER Overrepresentation Test (Released 20240807)					
Annotation Version and Release Date:	PANTHER version 19.0 Released 2024-06-20					
Analyzed List:	upload_1 (Mus musculus)					
Reference List:	Mus musculus (all genes in database)					
Test Type:	FISHER					
Correction:	BONFERRONI					
Bonferroni count:	1772					
PANTHER GO-Slim Biological Process	Mus musculus - REFLIST (21836)	upload_1 (382)	upload_1 (expected)	upload_1 (over/under)	upload_1 (fold Enrichment)	upload_1 (P-value)
receptor signaling pathway via JAK-STAT (GO:0007259)	11	7	0.19	+	36.38	2.61E-07
receptor signaling pathway via STAT (GO:0097696)	11	7	0.19	+	36.38	2.61E-07
intracellular receptor signaling pathway (GO:0030522)	14	8	0.24	+	32.66	3.96E-08
calcineurin-mediated signaling (GO:0097720)	10	5	0.17	+	28.58	6.63E-04
cytokine production (GO:0001816)	10	5	0.17	+	28.58	6.63E-04
hormone-mediated signaling pathway (GO:0009755)	28	9	0.49	+	18.37	1.27E-06
anterior/posterior pattern specification (GO:0009952)	46	13	0.8	+	16.15	1.26E-09
sensory organ development (GO:0007423)	66	18	1.15	+	15.59	8.92E-14
**regulation of neurogenesis (GO:0050767)**	45	12	0.79	+	15.24	2.10E-08
regionalization (GO:0003002)	53	13	0.93	+	14.02	9.29E-09
regulation of cell development (GO:0060284)	68	15	1.19	+	12.61	1.13E-09
regulation of transcription by RNA polymerase II (GO:0006357)	1565	334	27.38	+	12.2	0.00E00
pattern specification process (GO:0007389)	66	14	1.15	+	12.13	1.19E-08
positive regulation of transcription by RNA polymerase II (GO:0045944)	229	45	4.01	+	11.23	8.06E-31
regulation of nervous system development (GO:0051960)	62	12	1.08	+	11.06	1.21E-06
regulation of DNA-templated transcription (GO:0006355)	1839	344	32.17	+	10.69	0.00E00
regulation of RNA biosynthetic process (GO:2001141)	1840	344	32.19	+	10.69	0.00E00
regulation of RNA metabolic process (GO:0051252)	1999	344	34.97	+	9.84	7.31E-316
regulation of nucleobase-containing compound metabolic process (GO:0019219)	2061	345	36.06	+	9.57	6.20E-313
positive regulation of DNA-templated transcription (GO:0045893)	279	45	4.88	+	9.22	6.52E-27
positive regulation of RNA biosynthetic process (GO:1902680)	279	45	4.88	+	9.22	6.52E-27
**regulation of cell differentiation (GO:0045595)**	112	18	1.96	+	9.19	1.78E-09
regulation of gene expression (GO:0010468)	2347	347	41.06	+	8.45	6.35E-296
regulation of macromolecule biosynthetic process (GO:0010556)	2370	347	41.46	+	8.37	2.37E-294
regulation of cellular biosynthetic process (GO:0031326)	2386	347	41.74	+	8.31	2.87E-293
regulation of biosynthetic process (GO:0009889)	2392	347	41.85	+	8.29	7.28E-293
response to peptide hormone (GO:0043434)	49	7	0.86	+	8.17	3.84E-02
negative regulation of transcription by RNA polymerase II (GO:0000122)	182	26	3.18	+	8.17	2.88E-13
regulation of nitrogen compound metabolic process (GO:0051171)	2449	345	42.84	+	8.05	3.39E-285
response to peptide (GO:1901652)	50	7	0.87	+	8	4.40E-02
regulation of primary metabolic process (GO:0080090)	2479	345	43.37	+	7.96	2.95E-283
positive regulation of RNA metabolic process (GO:0051254)	331	45	5.79	+	7.77	1.19E-23
cellular response to hormone stimulus (GO:0032870)	96	13	1.68	+	7.74	2.26E-05
response to hormone (GO:0009725)	121	16	2.12	+	7.56	6.72E-07
regulation of cellular metabolic process (GO:0031323)	2674	347	46.78	+	7.42	5.04E-275
positive regulation of nucleobase-containing compound metabolic process (GO:0045935)	357	46	6.25	+	7.37	3.28E-23
regulation of macromolecule metabolic process (GO:0060255)	2725	347	47.67	+	7.28	5.11E-272
head development (GO:0060322)	63	8	1.1	+	7.26	2.42E-02
**brain development (GO:0007420)**	63	8	1.1	+	7.26	2.42E-02
regulation of metabolic process (GO:0019222)	2870	347	50.21	+	6.91	8.58E-264
regulation of cytokine production (GO:0001817)	86	10	1.5	+	6.65	4.59E-03
**cell differentiation (GO:0030154)**	859	98	15.03	+	6.52	7.85E-49
cellular developmental process (GO:0048869)	859	98	15.03	+	6.52	7.85E-49
embryo development (GO:0009790)	88	10	1.54	+	6.5	5.68E-03

positive regulation of macromolecule biosynthetic process (GO:0010557)	402	45	7.03	+	6.4	4.45E-20
positive regulation of cellular biosynthetic process (GO:0031328)	409	45	7.16	+	6.29	9.08E-20
positive regulation of biosynthetic process (GO:0009891)	410	45	7.17	+	6.27	1.00E-19
negative regulation of DNA-templated transcription (GO:0045892)	237	26	4.15	+	6.27	1.81E-10
negative regulation of RNA biosynthetic process (GO:1902679)	238	26	4.16	+	6.24	2.00E-10
animal organ morphogenesis (GO:0009887)	102	11	1.78	+	6.16	3.09E-03
regulation of multicellular organismal development (GO:2000026)	142	15	2.48	+	6.04	5.41E-05
negative regulation of RNA metabolic process (GO:0051253)	257	26	4.5	+	5.78	1.21E-09
regulation of cell population proliferation (GO:0042127)	173	16	3.03	+	5.29	1.24E-04
developmental process (GO:0032502)	1411	130	24.68	+	5.27	2.98E-56
negative regulation of nucleobase-containing compound metabolic process (GO:0045934)	284	26	4.97	+	5.23	1.20E-08
**axon development (GO:0061564)**	151	13	2.64	+	4.92	4.72E-03
animal organ development (GO:0048513)	434	37	7.59	+	4.87	3.42E-12
positive regulation of nitrogen compound metabolic process (GO:0051173)	541	46	9.46	+	4.86	1.12E-15
regulation of multicellular organismal process (GO:0051239)	328	27	5.74	+	4.71	5.54E-08
positive regulation of cellular metabolic process (GO:0031325)	581	46	10.16	+	4.53	1.83E-14
**neuron differentiation (GO:0030182)**	306	24	5.35	+	4.48	1.89E-06
positive regulation of macromolecule metabolic process (GO:0010604)	589	46	10.3	+	4.46	3.12E-14
anatomical structure morphogenesis (GO:0009653)	436	34	7.63	+	4.46	6.52E-10
regulation of developmental process (GO:0050793)	251	19	4.39	+	4.33	1.86E-04
**generation of neurons (GO:0048699)**	319	24	5.58	+	4.3	4.33E-06
anatomical structure development (GO:0048856)	1252	89	21.9	+	4.06	1.68E-27
positive regulation of metabolic process (GO:0009893)	659	46	11.53	+	3.99	2.24E-12
**neurogenesis (GO:0022008)**	348	24	6.09	+	3.94	2.37E-05
**neuron development (GO:0048666)**	233	16	4.08	+	3.93	6.77E-03
response to endogenous stimulus (GO:0009719)	237	16	4.15	+	3.86	8.42E-03
regulation of cellular process (GO:0050794)	5475	351	95.78	+	3.66	3.52E-170
negative regulation of nitrogen compound metabolic process (GO:0051172)	425	26	7.43	+	3.5	6.76E-05
multicellular organism development (GO:0007275)	819	50	14.33	+	3.49	2.38E-11
regulation of biological process (GO:0050789)	5834	353	102.06	+	3.46	1.54E-163
negative regulation of macromolecule biosynthetic process (GO:0010558)	434	26	7.59	+	3.42	1.03E-04
negative regulation of biosynthetic process (GO:0009890)	438	26	7.66	+	3.39	1.23E-04
negative regulation of cellular biosynthetic process (GO:0031327)	438	26	7.66	+	3.39	1.23E-04
system development (GO:0048731)	675	39	11.81	+	3.3	1.24E-07
biological regulation (GO:0065007)	6133	354	107.29	+	3.3	2.13E-157
**nervous system development (GO:0007399)**	468	27	8.19	+	3.3	1.23E-04
negative regulation of cellular metabolic process (GO:0031324)	537	26	9.39	+	2.77	5.86E-03
negative regulation of macromolecule metabolic process (GO:0010605)	587	26	10.27	+	2.53	2.82E-02
positive regulation of cellular process (GO:0048522)	1180	51	20.64	+	2.47	5.05E-06
negative regulation of metabolic process (GO:0009892)	606	26	10.6	+	2.45	4.85E-02
positive regulation of biological process (GO:0048518)	1383	52	24.19	+	2.15	3.41E-04
multicellular organismal process (GO:0032501)	1760	58	30.79	+	1.88	4.58E-03
biological_process (GO:0008150)	13046	361	228.23	+	1.58	8.82E-53
cellular component organization (GO:0016043)	2220	13	38.84	-	0.33	1.51E-03
cellular component organization or biogenesis (GO:0071840)	2337	13	40.88	-	0.32	2.91E-04
cellular biosynthetic process (GO:0044249)	1734	7	30.33	-	0.23	4.92E-04
organic substance biosynthetic process (GO:1901576)	1778	7	31.1	-	0.23	2.42E-04
biosynthetic process (GO:0009058)	1782	7	31.17	-	0.22	2.42E-04
cellular metabolic process (GO:0044237)	3153	8	55.16	-	0.15	1.92E-13
macromolecule metabolic process (GO:0043170)	2903	7	50.79	-	0.14	2.69E-12
Unclassified (UNCLASSIFIED)	8790	21	153.77	-	0.14	0.00E00
nucleic acid metabolic process (GO:0090304)	902	2	15.78	-	0.13	4.19E-02
organic substance metabolic process (GO:0071704)	3947	8	69.05	-	0.12	1.08E-19
metabolic process (GO:0008152)	4181	8	73.14	-	0.11	8.90E-22
nucleobase-containing compound metabolic process (GO:0006139)	1130	2	19.77	-	0.1	9.81E-04
heterocycle metabolic process (GO:0046483)	1187	2	20.77	-	0.1	4.76E-04
cellular aromatic compound metabolic process (GO:0006725)	1195	2	20.91	-	0.1	3.10E-04

analysis

organic cyclic compound metabolic process (GO:1901360)	1284	2	22.46	-	0.09	6.59E-05
cellular nitrogen compound metabolic process (GO:0034641)	1349	2	23.6	-	0.08	3.24E-05
transport (GO:0006810)	1694	2	29.63	-	0.07	5.00E-08
establishment of localization (GO:0051234)	1792	2	31.35	-	0.06	9.78E-09
localization (GO:0051179)	1981	2	34.66	-	0.06	3.59E-10
primary metabolic process (GO:0044238)	3509	3	61.39	-	0.05	8.79E-22
nitrogen compound metabolic process (GO:0006807)	3166	2	55.39	-	0.04	4.27E-20
